# The Neurobiology of Fear Generalization

**DOI:** 10.3389/fnbeh.2018.00329

**Published:** 2019-01-15

**Authors:** Arun Asok, Eric R. Kandel, Joseph B. Rayman

**Affiliations:** ^1^Jerome L. Greene Science Center, Department of Neuroscience, Columbia University, New York, NY, United States; ^2^Zuckerman Mind Brain Behavior Institute, Columbia University, New York, NY, United States; ^3^Howard Hughes Medical Institute (HHMI), Columbia University, New York, NY, United States; ^4^Kavli Institute for Brain Science, Columbia University, New York, NY, United States

**Keywords:** fear generalization, fear memory, neural circuits, animal models, sex differences

## Abstract

The generalization of fear memories is an adaptive neurobiological process that promotes survival in complex and dynamic environments. When confronted with a potential threat, an animal must select an appropriate defensive response based on previous experiences that are not identical, weighing cues and contextual information that may predict safety or danger. Like other aspects of fear memory, generalization is mediated by the coordinated actions of prefrontal, hippocampal, amygdalar, and thalamic brain areas. In this review article, we describe the current understanding of the behavioral, neural, genetic, and biochemical mechanisms involved in the generalization of fear. Fear generalization is a hallmark of many anxiety and stress-related disorders, and its emergence, severity, and manifestation are sex-dependent. Therefore, to improve the dialog between human and animal studies as well as to accelerate the development of effective therapeutics, we emphasize the need to examine both sex differences and remote timescales in rodent models.

## Introduction

Fear is a primitive emotion that is conserved throughout the animal kingdom (Walters et al., 1981; LeDoux, 2012; Adolphs, 2013). Survival in the wild is critically dependent on the flexible assessment of threatening stimuli, which entails the processing, integration, and synthesis of information acquired by multiple sensory modalities. Because aversive experiences are never completely identical, animals must generalize their fear of a past experience to future encounters that bear a sufficient degree of similarity to the original event. Like other memory-related processes, generalization is modulated by a number of intrinsic factors, including internal states (estrous and circadian cycles; Hull, 1943; Toufexis et al., 2007; Koch et al., 2017), previous experience (Lashley and Wade, 1946), genetic background (Temme et al., 2014), and sex differences (Day et al., 2016; Keiser et al., 2017). Generalization is also influenced by external factors including the type and intensity of aversive stimulation (Baldi et al., 2004), early-life stress (Elliott and Richardson, 2018), as well as the saliency of particular elements in the environment (Huckleberry et al., 2016). Finally, generalization is sensitive to the passage of time, as memories naturally lose both their precision and strength (McAllister and McAllister, 1963; Winocur et al., 2007; Jasnow et al., 2012; Pollack et al., 2018). Given the large number of variables that impinge on the generalization of fear, it has been challenging to develop an overarching neurobiological framework with robust explanatory power. However, recent studies have begun to provide some compelling new insights. Furthermore, whereas generalization has adaptive value, overgeneralization is maladaptive, and is a major feature of anxiety- and stress-related disorders such as post-traumatic stress disorder (PTSD; Elzinga and Bremner, 2002; Lissek et al., 2010; Dunsmoor and Paz, 2015). Therefore, a better understanding of the neurobiology of generalization is essential from a translational perspective.

In this review article, we explore the neurobiology of fear generalization within a broader historical, theoretical, and behavioral context. We then outline how the neural circuits involved in fear generalization may shift with the passage of time. Finally, we examine our current understanding of the neurotransmitter systems and cellular signaling pathways that contribute to fear generalization, and discuss how this information may be used to develop new therapeutic approaches for treating disorders of fear memory.

## Adaptive vs. Maladaptive Fear Generalization

What defines the boundary between adaptive and maladaptive fear generalization? From an ethological perspective, generalized responses that promote survival of an organism are defined as adaptive, whereas behaviors that contradict the mandate of self-preservation are maladaptive (Johnson et al., 1992; McEwen, 1998; Cooper and Blumstein, 2015). However, this delineation must be qualified by several caveats.

First, the environmental context in which a generalized fear response occurs is a critical parameter, because a behavior that is adaptive in one environment may be maladaptive in another. For example, increased defensive behaviors and a reduction of foraging in areas of high predatory threat are adaptive for rodents. However, deployment of an enhanced defensive response in environments lacking an elevated imminence of threat is maladaptive because it unnecessarily compromises both the acquisition of resources and allostasis, which refers to the set of adaptive processes that maintain homeostasis (Fanselow, 1994; McEwen, 1998; Blanchard and Blanchard, 2008). The same inference can be drawn for humans as well as for laboratory mice, where test subjects that are conditioned in a particular context or to a particular cue generalize fear in different contexts or to different cues (Kaczkurkin et al., 2016), but see (Elzinga and Bremner, 2002). Cues and environments exist on a perceptual continuum, and maladaptive fear generalization occurs when an abnormal stimulus-response gradient emerges to produce defensive behaviors in environments or to cues which have never been explicitly associated with threat or danger.

In addition, sexually dimorphic generalization may serve an equally adaptive function within each sex for various behaviors (Darwin, 1888; Kelley, 1988). With regard to fear generalization, female mice that have been exposed to contextual fear conditioning tend to freeze in the first retrieval context in which they are tested, whether or not it is identical to the training context (Keiser et al., 2017). One possible interpretation of this behavior is that the consequence of making a “mistake” (i.e., not exhibiting an optimal defensive strategy) in a potentially life-threatening environment is evolutionarily more costly for female mice in terms of future reproductive success (Kelley, 1988). However, this example also illustrates that the evolutionary benefit of a given behavioral pattern is not definitively clear (for review see Bangasser and Wicks, 2017). Finally, although a particular behavior may be maladaptive for an individual it may actually benefit the population (for review see Miller and Polack, 2018).

For these reasons, it is not possible to demarcate adaptive and maladaptive behavior in absolute terms. Therefore, we favor a normative definition in which performance of sex-matched, wild-type animals in a given behavioral task serves as a reference for what constitutes adaptive behavior, with phenotypic outliers representing maladaptive states. Other research groups have sought to formalize the identification of maladaptive generalization states by stratifying animal behavior across a variety of behavioral paradigms (Cohen et al., 2003, 2004; Cohen and Zohar, 2004; Richter-Levin et al., 2018). As more studies begin to implement this strategy, a major challenge will be to establish agreed upon criteria for clearly defining the boundaries that separate normal from pathological fear generalization.

## Theoretical Framework

For well over a century, research has examined the behavioral correlates of stimulus generalization and discrimination. In the 1920s, the seminal studies of Pavlov demonstrated that animals trained in an auditory conditioning paradigm exhibit generalization of their conditioned response (CR) to a range of auditory stimuli (Pavlov, 1927). Subsequent work suggested that a failure to discriminate between the conditioned stimulus (CS) and similar, but non-identical stimuli is a result of: (1) an active process of inhibitory weakening (Spence, 1936); (2) the failure to form a strong association between the CS and unconditioned stimulus (US), indicating that the “dimensions” of a stimulus are not well-learned (Lashley and Wade, 1946; Rescorla and Wagner, 1972); and (3) forgetting, or the failure of retrieval (Bouton et al., 1999). Although generalization likely arises from a weighted sum of these processes, many of the studies covered in this review article have explored generalization within the boundaries of each independently. For example, changes in several brain regions have been shown to actively promote or inhibit discrimination (Duvarci et al., 2009; Cullen et al., 2015; Ferrara et al., 2017). Moreover, generalization can be partially alleviated by greater learning about the CS (Biedenkapp and Rudy, 2007; but see Poulos et al., 2016). However, the neurobiological contributions of “forgetting” to generalization are more difficult to evaluate [(Rescorla, 1976; Pearce, 1987; Riccio et al., 1992), but see (Ishikawa et al., 2016; Richards and Frankland, 2017)].

Our understanding of the neurobiology of fear generalization within the aforementioned theoretical constructs is further complicated by the temporal evolution of associative memories, whereby memories become less precise and rely more heavily on cortical areas over time (Bergstrom, 2016; Jasnow et al., 2017; Sekeres et al., 2017; Asok et al., 2018b). When considering these temporal factors, we are left with a challenging question: what are the neural and molecular mechanisms that control the generalization of fear memories at remote timescales? A number of conceptual frameworks originally developed to explain the shift of associative memories from limbic to cortical structures have also been applied to generalization. In particular, three key theories have prevailed: systems consolidation theory, multiple trace theory, and trace transformation theory.

In *systems consolidation theory*, episodic memories are transferred to the neocortex from the hippocampus, such that the expression of remote memories may no longer be hippocampus-dependent (Dudai, 2004; Dudai et al., 2015). However, a number of studies have challenged this view by showing that the hippocampus continues to play a role in the retrieval of remote fear memories (Rekkas and Constable, 2005; Lehmann et al., 2007; Clark and Sutherland, 2013). Moreover, neocortical areas may also be recruited during initial consolidation, though in an immature form (Zhao et al., 2005; Takehara-Nishiuchi et al., 2006; Vetere et al., 2011; Kitamura et al., 2017), a concept that other theoretical frameworks have attempted to incorporate (Asok et al., 2018b).

According to *multiple trace theory* (Moscovitch and Nadel, 1999; Moscovitch et al., 2005), neocortical and hippocampal areas are rapidly recruited to a memory trace, but these memories become less detailed and accurate over time. However, the act of retrieval produces a new memory trace and serves to strengthen hippocampal and neocortical connections as well as strengthen the overall memory (Moscovitch et al., 2005). Likewise, the *transformation hypothesis* suggests that context-specific episodic memory is always hippocampus-dependent, but details are lost over time as a particular memory becomes more schematic (see Broadbent and Clark, 2013). However, the transformed schematic representation is less precise and relies less on the hippocampus. Indeed, certain features of a memory persist longer than others and are differentially consolidated across the brain (Malin and McGaugh, 2006; Wiltgen et al., 2010). Thus, while certain brain areas may be especially suited for encoding specific aspects of a fear memory [e.g., foot-shock or context (Malin and McGaugh, 2006)], the subsequent retrieval of the memory may rely more heavily on another set of brain regions at recent vs. remote time points (Frankland et al., 2004).

These theories of long-term memory outlined above are, however, limited in their ability to provide a comprehensive framework for understanding fear generalization. For example, what we know about remote episodic memory is largely predicated on hippocampal-based mechanisms, despite the fact that generalization clearly involves the amygdala, frontal cortex, and other brain regions. Along these lines, these theories of long-term memory also do not embrace the fundamental circuit-wide nature of memory and generalization at recent vs. remote timescales, which may be completely independent of the hippocampus. Finally, and perhaps most important, these theories do not fully explain how memory becomes less precise over time (Bouton et al., 1999; Wiltgen and Silva, 2007), an issue that would need to be addressed by any robust theoretical model of generalization.

## Methodological Approaches in the Study of Fear Generalization

Although there is considerable variation in methodology across studies, behavioral studies in rodents have focused on generalization to either contextual or discrete cues. In context generalization experiments, a rodent is typically exposed to contextual fear conditioning, which entails the presentation of an aversive US such as a foot shock in a conditioning context—a previously neutral environment. Subsequent re-exposure of the animal to the conditioning context (CTX+) without delivery of the US evokes a species-specific defensive reaction such as freezing, which refers to the cessation of all movement except for respiration, and is generally accepted as a proxy for fear (Blanchard and Blanchard, 1969). In turn, freezing in the CTX+ in the absence of a US can be measured at various time points after the initial CS-US pairing. When assessed at 24 h, which is perhaps the most commonly used interval of time in these experiments, freezing is an index of long-term associative memory, with longer intervals (several weeks or longer) corresponding to remote associative memory.

To measure contextual fear generalization, animals are fear conditioned and then exposed to a different context that was never paired with a shock (CTX−; Rohrbaugh and Riccio, 1968; Ruediger et al., 2011). Freezing in the CTX− is an index of fear generalization and can also be evaluated at multiple time intervals, although the degree to which the CTX+ and CTX− environments share similarities (e.g., odor, lighting, and chamber shape) can vary substantially between studies, and can greatly impact experimental outcomes, as we discuss later. Furthermore, recent work has found that similarity between olfactory and tactile elements of the CTX+ and CTX− are more important than visual cues for generalization in males relative to females [(Huckleberry et al., 2016), but see (Bucci et al., 2002; Murawski and Asok, 2017)]. Less is known about whether particular stimulus elements are more critical in females (e.g., odors given maternal roles), but within a species the most salient sensory elements are likely similar between males and females (Dunsmoor et al., 2011; Lissek et al., 2013).

**Figure 1:**
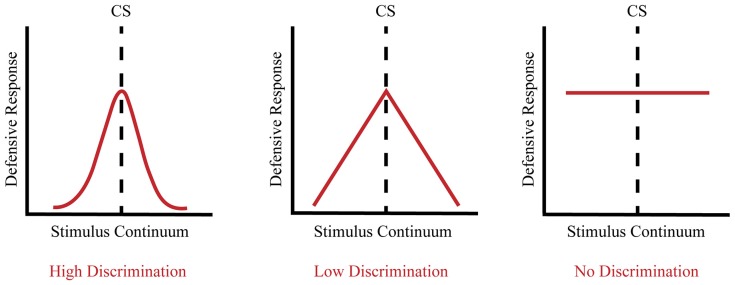
Fear generalization occurs along a continuum. High discrimination is a product of a heightened defensive response to the conditioned stimulus (CS) and a low defensive response to non-target CSs, reflecting a narrow generalization gradient (left panel). Low discrimination is a product of a heightened defensive response to the CS as well as an elevated defensive response to stimuli that approximate the CS, reflecting a broad generalization gradient (middle panel). No discrimination is a product of a heightened defensive response to the CS as well as stimuli that markedly differ from the CS, reflecting an elevated flat generalization gradient (right panel).

In contrast, fear generalization to discrete cues commonly involves exposing animals to an aversive US paired with a stimulus presented through one sensory modality such as a neutral tone or odor, which then becomes a CS. When subsequently presented with a cue that resembles the CS, animals exhibit a defensive response whose magnitude with respect to the original CR is dependent upon the perceptual similarity of the two stimuli (Shaban et al., 2006; Zhang et al., 2017). In psychometric terms, the strength of the defensive response varies as a function of the degree to which the new CS approximates the original CS (see Figure 1). Thus, a narrow generalization gradient (high discrimination) is signified by a maximal defensive response that only occurs within a narrow range of stimuli that are very similar to the CS, whereas a broad generalization gradient (low discrimination) is indicated by the ability of progressively dissimilar stimuli to elicit a defensive response. It is important to note that the type of conditioning (e.g., auditory trace fear conditioning vs. unpaired controls) can influence generalization. Given that discrete cues are always presented in a particular context, the type of conditioning can influence both the associative value of the CS and the associative value of the context. Thus, discrete cue conditioning paradigms that manipulate how the context is presented, either in the foreground or in the background of the discrete cue, can differentially influence fear discrimination (Rescorla, 1976; Pearce, 1987; Desmedt et al., 2003).

It is more straightforward to parametrically examine generalization using a discrete cue vs. a context. For example, one can alter the frequency of a tone by defined gradations and observe an animal’s response during different cue presentations (Guttman and Kalish, 1956). In an analogous manner, a structurally related series of odorants that differ by a single carbon group can produce a generalization gradient (Pavesi et al., 2013). This is not as easily accomplished in contextual generalization experiments, because contextual representations reflect a combination of stimulus elements (e.g., spatiotemporal elements, as well as tactile, olfactory, visual, and auditory inputs) that are bound into a unitary representation (Sutherland and Rudy, 1989; O’Reilly and Rudy, 2001; Rudy, 2009). Furthermore, given the number of potentially salient features in a contextual fear conditioning chamber, the extent to which an animal may attend to one element over another is poorly understood, although computational models of serial element processing have been proposed (Krasne et al., 2015).

Yet, in both types of generalization, behavior can be modulated by a number of external factors, including strength and duration of the US, strength of the CS-US association, similarity between the CS and generalization stimuli, as well as a number of internal factors such as genetic background, sex, and circadian cycle. Moreover, these parameters can interact with one another. For example, pre-exposing male mice to a conditioning context (CTX+) can enhance the strength of recent fear memories during single-trial conditioning (McHugh and Tonegawa, 2007; Brown et al., 2011), but it also produces generalization to the CTX− (Radulovic et al., 1998; Rudy and O’Reilly, 1999). In addition, whereas pre-exposing females to the CTX+ reduces generalization without altering the strength of recent fear memories, pre-exposing males to the CTX− enhances generalization to the pre-exposed context (CTX−; Keiser et al., 2017). These observations are further complicated by the fact that generalization is dependent on the test order of the different contexts, whereby extinction produced by testing in an non-reinforced context may influence the generalization of fear in a subsequent test context (Wood and Anagnostaras, 2011; Huckleberry et al., 2016; Keiser et al., 2017).

The diverse behavioral outputs observed in the latter experiments are ostensibly a reflection of adaptive tuning mechanisms that are modulated by a number of critical parameters, including animal species and strain. Furthermore, it is important to emphasize the potential contributions of sex differences. For example, when considering external factors that contribute to fear generalization in males relative to females the recruitment of different brain regions (e.g., amygdala vs. hippocampus) may be an important variable (Keiser et al., 2017). In addition, new studies are beginning to identify how active or passive defense strategy selection may differ between sexes (Gruene et al., 2015; Shansky, 2018). It is possible that ovarian hormonal state in females may alter the functional connectivity of neural circuits during specific temporal windows, leading to differential effects on stress reactivity and memory (Andreano et al., 2018), which in turn could affect generalization. Regardless of the range of parametric factors that mediate the generalization of recently acquired fear memories, animals may generalize their fear to the CTX− at remote time-points (Balogh et al., 2002; Wiltgen and Silva, 2007; Poulos et al., 2016; Pollack et al., 2018; but see Biedenkapp and Rudy, 2007; Vanvossen et al., 2017). Despite methodological differences across studies, sex differences in generalization at recent and remote time-points, whether to cues or contexts, are likely a product of alterations in information processing within fear circuits. Cellular and molecular changes within these neural circuits that control normal fear learning and memory likely serve as key conduits for promoting or inhibiting fear generalization between sexes across time.

## Neural Circuits of Fear Generalization

Fear memories rely on discrete neural circuits which shift as a function of the type of CS-US pairing (e.g., discrete cues in trace or delay conditioning vs. contextual conditioning; for review see Maren, 2001; Tovote et al., 2015). US foot-shock information from peripheral sensory inputs enter the ventroposterior nucleus of the thalamus (VPN) as well as the posterior intralaminar nucleus of the thalamus (PIN). Accordingly, studies have found that electrolytic lesions of the PIN disrupt fear conditioning (Lanuza et al., 2004, 2008). US information from the PIN and the posterior insular cortex (PIC) is then relayed to the lateral nucleus of the amygdala (LA), which is a critical site of plasticity in fear learning and memory regardless of the type of CS-US pairing (Goosens and Maren, 2001). However, US pathways also show selectivity for the type of CS-US pairing in that lesions of the PIC only disrupt auditory, but not contextual, fear memories (Brunzell and Kim, 2001; Davis, 2006), which likely reflects multimodal information processing.

### Auditory Fear Circuits

During auditory fear conditioning, auditory information is relayed from lemniscal and extralemniscal pathways to the auditory thalamus. This information from the auditory thalamus is then relayed to the LA by either a direct pathway arising from extralemniscal projections originating in the medial part of the medial geniculate nucleus (mMGN) and PIN, or by an indirect pathway, which arises out of the lemniscal pathway and projects from the ventral MGN to the primary auditory cortex, and subsequently to the auditory association cortex and then LA (Weinberger, 2011). Inputs to the LA from the mMGN are critical for fear memories and, as discussed later, molecular perturbations in the mMGN produce fear generalization (Nabavi et al., 2014; Ferrara et al., 2017). Auditory CS and US foot-shock information is thought to converge in the LA and in parts of the central nucleus of the amygdala (CeA; Paré et al., 2004). The Excitatory and inhibitory balance of discrete populations of neurons in the LA for tones, and basal amygdala complex for contexts given inputs from the ventral hippocampus (Canteras and Swanson, 1992; Maren and Fanselow, 1995), has been implicated in fear generalization as similarity of the CS− approaches the CS+ (Tovote et al., 2015; Rajbhandari et al., 2016; Grosso et al., 2018). The LA provides inputs to the CeA, of which the medial division (CeAm) contains the primary outputs to structures which mediate behavioral and neuroendocrine aspects of fear (e.g., the periaqueductal gray; PAG; Gross and Canteras, 2012). Interestingly the lateral division of the CeA (CeAl) also receives direct inputs from the thalamus (Linke et al., 2000), provides tonic inhibition of the CeAm, and is associated with fear generalization to auditory CSs (Ciocchi et al., 2010). Moreover, recent studies have suggested that corticotropin releasing factor in the CeAl may be important for modulating fear generalization under conditions of low-associative strength (Sanford et al., 2017). However, the CeAl and basolateral amygdala (BLA) complex also send projections to the bed nucleus of the stria terminalis (BNST), a region implicated in anxiety-like behaviors and contextual fear (Dong et al., 2001; Davis et al., 2010; Asok et al., 2018a). Lesions of the BNST enhance the precision of recent auditory memories while reducing fear generalization (Duvarci et al., 2009).

### Contextual and Olfactory Fear Circuits

The dorsal hippocampus is critical for the formation of a unitary contextual representation during contextual fear conditioning (Maren et al., 1997; Holland and Bouton, 1999; Rudy et al., 2004). Information from sensory and association cortices is relayed to post-rhinal (POR) and peri-rhinal (PER) cortices, followed by the medial and lateral entorhinal cortices [MEC and LEC, respectively (Lee and Lee, 2013)]. This information from different MEC and LEC layers then flows into the hippocampal formation, with segregated inputs to the dorsal dentate gyrus, dorsal hippocampal CA3 subfield, dorsal CA1 subfield, and dorsal subiculum (dSub). Indeed, distinct outputs from CA1 and dSub to MEC are important for the acquisition and retrieval of recent fear memories, respectively (Roy et al., 2017). The DG and CA3 have received considerable attention for their role in fear generalization because of their contributions to pattern separation and pattern completion (see McHugh et al., 2007; Rolls, 2013). Mice with deletion of the N-methyl-D-aspartate receptor (NMDAR) in CA3 exhibit generalization during short-term, but not recent, fear memory tests, suggesting that CA3 has an important role in the rapid formation of contextual representations (Cravens et al., 2006). Recent studies have suggested that while neuronal ensembles in the DG show context selectivity during the retrieval of recent fear memories, there is a substantial loss of DG selectively at remote time-points which parallels fear generalization (Matsuo, 2015; Yokoyama and Matsuo, 2016). It is worth noting that the DG is one of the few sites in the brain that exhibits neurogenesis, and manipulations that promote neurogenesis improve contextual discrimination—a finding which suggests that enhancing DG function may increase remote memory precision (Sahay et al., 2011; Nakashiba et al., 2012; Besnard and Sahay, 2016).

Given the importance of olfactory cues to rodents and their relevance to disorders such as PTSD (Rolls et al., 2013; Cortese et al., 2015), as well as the influence of neurogenesis in the olfactory bulb, it will also be interesting to determine whether manipulating neurogenesis in the olfactory bulb modulates fear generalization to odors (see Tong et al., 2014).

### Hippocampal-Thalamic-Prefrontal Circuits

The functional distinction between the dorsal hippocampus and ventral hippocampus has long-been debated, but there is agreement that both divisions are essential in the consolidation of contextual fear memories (Fanselow and Dong, 2010; Zhu et al., 2014). Moreover, the ventral hippocampus and its connections may be important for the maintenance of memory precision (Ciocchi et al., 2015; Cullen et al., 2015; Jimenez et al., 2018). The ventral hippocampus has reciprocal connections with the medial prefrontal cortex (mPFC; anterior cingulate, prelimbic, and infralimbic regions), the BLA, the retrosplenial cortex, and the insular cortices (Pitkänen et al., 2000; Cenquizca and Swanson, 2007). Interestingly, neuronal activity in the mPFC increases in parallel with the emergence of fear generalization at remote time-points (Cullen et al., 2015), suggesting the possibility of an active role in promoting generalization. Moreover, the mPFC is reciprocally linked with vCA1 by the nucleus reuniens (NR), and enhancing activity of mPFC inputs to the NR decreases contextual fear generalization (Xu and Südhof, 2013). Thus, the mPFC ↔ NR ↔ vCA1 circuit is likely to play an important role in the modulation of memory precision as well as the generalization of fear during the natural course of systems-consolidation (Rozeske et al., 2015; Ramanathan et al., 2018). In humans, other brain regions such as the striatum, insula, and PAG have also been implicated in the generalization of recent fear memories (Dunsmoor et al., 2011).

### Considerations in the Neural Circuits of Fear Generalization

Generalization gradients exist in core sensory cortices which process discrete CSs such as odors and tones. The generalization of contextual fear appears to follow a similar organizational structure, but involves a more elaborate network to account for multimodal sensory and representational processing. How fear generalization gradients emerge and shift across time at the neural circuit level is an important area of future research. For example, understanding how contextual information differentially engages the dorsal hippocampal, ventral hippocampal, and medial prefrontal circuits at recent and remote time-points may provide important insights into a global framework for how fear generalization to complex representations occurs. Similarly, identifying whether these shifts are paralleled in sensory cortices which represent the elemental components of a contextual representation, such as auditory information in direct and indirect thalamic relay pathways to the LA, will help to build a global framework of the brain-wide circuits which modulate fear generalization (Weinberger, 2011; Shang et al., 2015). However, an important consideration in this work is the interaction between remote fear generalization and systems consolidation. Beyond neural circuits, this interaction raises a fundamental biological question: are the molecular and genetic mechanisms that regulate fear generalization at early time-points similar to those at remote time-points?

While most of the neural circuits and molecular mechanisms enumerated above promote generalization, it is also clear that generalization is an active process in which a consolidated memory is prevented from undergoing accurate retrieval. For example, inactivation of the ACC or vCA1 has no effect on contextual fear memory at remote time-points when mice are tested in the training context, but enhanced freezing to a novel context is suppressed (Frankland et al., 2004; Cullen et al., 2015). Thus, the ability to discriminate between an aversive context and neutral context at remote time-points is actively inhibited by the ACC and vCA1. Similarly, blocking protein synthesis in the MGN enables tone discrimination that is otherwise not observed in an auditory fear conditioning paradigm (Ferrara et al., 2017). Also, as mentioned previously, lesions of the BNST reduce fear generalization (Duvarci et al., 2009), implying an unidentified role for this brain region and its outputs in the active suppression of discrimination.

Finally, the substrates of generalization are nested within the neural circuits that support fear memory. For example, the acquisition, extinction, and generalization of fear are all regulated by NMDARs functioning in excitatory neurons of the PFC (Vieira et al., 2015). It is therefore possible that experimental manipulations which target associative fear memories may exert uncharacterized effects on generalization, and would merit further exploration. However, the molecular and cellular mechanisms that support various components of associative fear memory including generalization are not entirely identical. For example, pharmaco-genetic deletion of a subset of excitatory and inhibitory neuronal ensembles in the amygdala impairs generalization, but not fear memory (Grosso et al., 2018), demonstrating that these processes are separable.

## Molecular and Cellular Mechanisms of Fear Generalization

Adding to the complexity of circuit-level processes are the many distinct molecular and cellular pathways that also contribute to generalization. These cellular and molecular pathways represent conduits for information that do not operate in isolation from one another. For example, the cannabinoid CB_1_ receptor is found in GABAergic and glutamatergic neurons in the CNS, thus allowing the endocannabinoid system to influence the activity of both inhibitory and excitatory synapses (Kano et al., 2009). Moreover, certain neurons may release both gamma-aminobutyric acid (GABA) and glutamate (Shabel et al., 2014), and changes in the excitatory and inhibitory balance within neurons may be important for certain behaviors (Froemke, 2015; Mongillo et al., 2018). Furthermore, signaling pathways intersect not only at the cellular level, but also at the circuit level. For instance, glucocorticoid and beta-adrenergic signaling across the limbic system cooperate in the regulation of long-term memory (Rodrigues et al., 2009; Roozendaal et al., 2009; McIntyre et al., 2012). Nevertheless, for organizational purposes and the sake of simplicity, we focus on particular contributions of discrete pathways.

### Excitatory and Inhibitory Neurotransmission

Activity within neural circuits involved in the storage and processing of memory is governed by a balance of excitatory and inhibitory neurotransmission (Froemke, 2015; Mongillo et al., 2018). Altering this balance impinges on circuit-level functions, leading to distinct alterations in behavioral outputs. Not surprisingly, in addition to their central roles in associative fear memory, both excitatory and inhibitory neurotransmission are also important in fear generalization.

Glutamate is the major excitatory neurotransmitter in the brain, and direct evidence for the role of glutamatergic signaling in fear generalization is provided by studies that target ionotropic glutamate receptors. The NMDAR, in particular, plays a key role in synaptic plasticity and memory (Tsien et al., 1996). Conditional deletion of an obligatory subunit (NR1) of the NMDAR in excitatory CaMKIIα-positive principle neurons within the PFC causes a time-dependent increase in generalization to auditory cues, which is driven by ineffective CS− learning (Vieira et al., 2015). Thus, NMDAR activation in excitatory neurons is critical for stimulus discrimination because it promotes a reduction in defensive behavior when an animal is presented with a non-reinforced CS−. In addition to mPFC-dependent mechanisms for generalization, glutamatergic signaling at excitatory NR1 subunit-containing NMDARs in the hippocampus is important for pattern separation and contextual fear memory (McHugh et al., 2007). Specifically, while contextual fear conditioning and discrimination between very different contexts is intact following NR1 deletion in dentate granule cells, KO mice cannot easily discriminate between perceptually similar contexts. Also, selective inactivation of NMDARs in the LA reveals a role for NMDA signaling in auditory fear generalization (Jones et al., 2015).

Complementing the latter studies, more recent work has found that injection of NMDA into the rodent prelimbic cortex to activate NMDARs during the consolidation or retrieval phases of contextual fear conditioning induces fear generalization (Vanvossen et al., 2017). However, the effect is only observed for strong fear conditioning, whereas NMDA injection during a weaker training protocol actually enhances contextual discrimination, a finding that is consistent with the idea that the mPFC is involved in promoting retrieval of weaker memories (Rudy et al., 2005). Thus, there exists a complex relationship between the magnitude of aversive stimuli and prelimbic mPFC activation with respect to different components of associative fear memory.

Although most studies that implicate glutamatergic signaling in fear generalization focus on NMDAR-dependent mechanisms, AMPA-dependent signaling is also important. For example, peptide-mediated blocking of the removal of AMPA receptors in the dorsal hippocampus maintains long-term contextual fear memory and inhibits generalization, which in turn correlates with inhibition of synaptic depotentiation (Migues et al., 2016). Another study found that upregulation of synaptic expression of GluR1-containing AMPA receptors in the amygdala may drive the generalization of auditory fear (Ferrara et al., 2017). Together, these studies point to an elementary role for glutamatergic signaling in both contextual and cued generalization, but also underscore the complex relationship between excitatory neurotransmission and behavior.

Counterbalancing the actions of glutamate is GABA, the major inhibitory neurotransmitter in the brain. GABAergic neurons in the amygdala and hippocampus play a critical role in the formation of fear memories (Fendt and Fanselow, 1999). Whereas ionotropic GABA_A_ receptors mediate fast inhibitory signaling, metabotropic GABA_B_ receptors exert a slow inhibitory tone over synaptic circuits (Chua and Chebib, 2017; Frangaj and Fan, 2018). Several lines of genetic evidence demonstrate that GABAergic transmission is involved in the generalization of both contextual and cued fear memory. For example, deletion of GABA_B(1a)_ receptors in mice is associated with increased contextual generalization, but has no impact on acquisition or maintenance of fear memory (Cullen et al., 2014). Likewise, deletion of the GABA_B_ receptor subtype leads to generalization of cued fear without affecting retrieval by CS+ presentation, an effect that is evident for high- but not low-intensity foot shocks (Shaban et al., 2006). Similarly, deletion of GABA_A_ receptor δ subunit causes an increase in generalization to auditory cues (Zhang et al., 2017). It will be interesting to see if perturbation of GABA_A_ receptors within discrete circuits also has an impact on the generalization of contextual fear. Finally, deletion of glutamic acid decarboxylase (GAD65), an enzyme responsible for synthesizing GABA, causes generalization to auditory cues, although GAD65 KO mice show normal contextual fear learning (Bergado-Acosta et al., 2008; Sangha et al., 2009). Together, these studies indicate that GABAergic transmission contributes to both cued and contextual fear generalization, but like glutamatergic signaling, the relationship between inhibitory neurotransmission and behavior is both subtle and complex.

### Monoaminergic Signaling

Among the classical monoamine neurotransmitters, dopamine appears to be the most significant modulator of fear generalization, although serotonin and noradrenaline (NA) can also regulate the processing of fear memory. Dopamine is critically involved in motivation, salience, reward learning, and prediction error (Schultz et al., 1997; Bromberg-Martin et al., 2010). In addition, a number of genetic and pharmacological studies have demonstrated a contribution for dopamine signaling in fear memory, which is mediated by dopamine receptors expressed in the hippocampus, amygdala, PFC, and striatum (Civelli et al., 1993; Pezze and Feldon, 2004). For example, mice lacking the dopamine D1 receptor (D1R) in granule cells of the dentate gyrus and the striatum exhibit poor retention of contextual fear (Ikegami et al., 2014; Sarinana et al., 2014). However, studies examining the impact of global D1R deletion on contextual fear memory have yielded mixed results (Ortiz et al., 2010; Abraham et al., 2016), which may be explained by methodological differences as well as the possibility of recruitment of compensatory pathways. Importantly, with respect to generalization, mice lacking D1R in the dentate gyrus are unable to discriminate between the training context and a novel context after exposure to contextual fear conditioning (Sarinana et al., 2014), while a modest increase in contextual fear generalization is observed when D1R is globally deleted (Abraham et al., 2016). Finally, cued fear memory may or may not be affected by D1R deletion (Ortiz et al., 2010; Sarinana et al., 2014; Abraham et al., 2016), which may be attributed to methodological differences in the studies. It remains to be seen whether these manipulations have an impact on generalization to discrete cues.

In contrast to D1R deletion, pharmacological studies reveal a role for dopamine D2 receptors (D2Rs) in cued fear generalization. For example, cannulated delivery of the D2R antagonist, raclopride, into the CeA or BNST is sufficient to increase generalization to auditory cues (De Bundel et al., 2016). Specifically, in the latter study, raclopride increases generalization to the CS− tone, while the dopamine receptor agonist, quinpirole, had the opposite effect. On the other hand, in a human fMRI study, pharmacological blockade of dopamine D2Rs produces a reduction in stimulus generalization (Kahnt and Tobler, 2016). Differences in drug specificity or route of delivery may explain these discordant effects. Nevertheless, the authors suggest that the hippocampus flexibly modulates the width of the stimulus generalization gradient, and that the hippocampus can provide active inhibition of generalization, by recruiting a dopamine-dependent process during retrieval. Given that the visual discrimination task employed in the latter study is based on positive reward, it would be interesting to evaluate the effects of D2R blockade in an aversive task in humans.

NA is a neurotransmitter involved in the consolidation of emotional memories during attentional processes, which in turn are essential for maintaining precision of memory (McGaugh, 2013). In rats exposed to contextual fear conditioning, pharmacological enhancement of noradrenergic transmission enhances the consolidation of memory, but also increases generalization of the freezing response to a neutral context (Gazarini et al., 2013). However, the latter effect on generalization was not observed when NA activity was induced after retrieval of fear memory, indicating an important role for NA presumably by interacting with stress hormones at the time of fear learning (McReynolds et al., 2010).

Finally, serotonin signaling is mediated by a large family of serotonin receptors and transporters that exerts complex, often paradoxical effects on both cued and contextual fear memory (Homberg, 2012; Burghardt and Bauer, 2013). However, far less is known about the impact of serotonergic signaling on generalization, which may reflect the complex relationship between serotonin and fear memory. One of the rare examples of research focusing on serotonin-dependent generalization found that male mice lacking the serotonin 1A receptor (5-HT1AR) exhibited heightened generalization of contextual fear, which is proposed to be a hippocampus-dependent effect (Klemenhagen et al., 2006).

### Hormones

Contextual generalization occurs more rapidly in female rats compared to males, and is partly mediated by estrogen (Lynch et al., 2013). In ovariectomized female rats, treatment with either an estrogen receptor (ER) agonist or estrogen itself enhances generalization to a neutral context in a passive avoidance task, an effect that is mediated by cytosolic or nuclear (but not membrane-bound) ERs in the dorsal hippocampus (Lynch et al., 2016b). These results provide yet another example of how retrieval of an aversive memory can be dissociated from generalization to a neutral context, because the latter manipulations that enhance generalization did not affect memory retrieval in the training context. Importantly, because the generalization of fear is associated with anxiety-related disorders, which have a disproportionately greater impact on women than men (Kessler et al., 2005, 2012; Tolin and Foa, 2006), estrogen-dependent mechanisms identified in rodents are likely to be clinically relevant. As with estrogen in females, testosterone in males is likewise capable of modulating the processing of fear memory. Gonadectomized male rats exhibit generalized fear to a neutral context in a passive avoidance task, which is ameliorated by injection with testosterone (Lynch et al., 2016a).

Corticosterone, the major glucocorticoid in rodents, is a steroid hormone that is released by the hypothalamic-pituitary-adrenal (HPA) axis during stress (de Kloet et al., 2005), and its effects on learning and memory are well documented (Schwabe et al., 2012; Meir Drexler and Wolf, 2017). In terms of fear generalization, a number of studies have implicated glucocorticoid-dependent signaling. For example, glucocorticoid receptors in the ventral hippocampus or BLA are important for contextual fear, and infusion of corticosterone into the hippocampus after fear conditioning prevents mice from discriminating between correct and incorrect predictors of threat (Donley et al., 2005; Kaouane et al., 2012). Although not all studies demonstrate an effect of corticosterone (Bueno et al., 2017), there is significant variation with regard to many key variables, including conditioning parameters, corticosterone regimen, species and strain differences, etc.

Finally, noradrenergic neurons in the locus coeruleus (LC) respond to orexin, a neuropeptide hormone produced by hypothalamic neurons and involved in the regulation of wakefulness, arousal, feeding behavior, and energy homeostasis (Sakurai, 2007). In conjunction with fear conditioning, optogenetic stimulation of orexinergic projections from the lateral hypothalamus to LC potentiates freezing to a novel context or cue (Soya et al., 2017). Furthermore, orexin neurons modulate a number of signaling pathways described above, including dopaminergic and cholinergic signaling (Sakurai, 2007).

### Transcriptional Regulatory Mechanisms

In addition to intercellular signaling, there are a number of intracellular, transcription-based mechanisms that contribute to fear generalization. For example, the cyclic-AMP response element binding (CREB) protein, an inducible transcription factor necessary for the consolidation of fear memories, has been shown to have regionally specific effects on fear generalization. Viral-based overexpression of CREB in the auditory thalamus not only enhances cued fear conditioning, but also increases generalization to the tone (Han et al., 2008). In the mPFC, depletion of the CREB binding protein (CBP), a transcriptional coactivator of CREB and histone acetyltransferase, reduces memory precision and enhances generalization of recent auditory fear memories (Vieira et al., 2014). Interestingly, this generalization emerges after discrimination training, which is consistent with the view that prefrontal circuits may have multiple roles across time (Frankland et al., 2004; Malin and McGaugh, 2006). More recently, the transcription factor Klf9 has been implicated as a stress- and sex-dependent regulator of fear generalization in male mice (Besnard et al., 2018).

### Other Signaling Pathways

Finally, limited evidence has supported a role for an assortment of other cellular signaling pathways in the generalization of fear, including nitric oxide, endocannabinoid, as well as cholinergic and neuropeptide Y (NPY) systems. For example, nitric oxide deficiency caused by deletion of the neuronal isoform of nitric oxide synthase (nNOS) increases generalization to odor in both male and female mice, and also inhibits olfactory fear memory (Pavesi et al., 2013). Activation of the cannabinoid system by cannabidiol (CBD) treatment in rodents has no impact on explicit contextual fear memory at 24 h, but generalization to a distinct context is significantly reduced (Stern et al., 2017). Pharmacological targeting of muscarinic acetylcholine receptors modulates generalization of fear with respect to an odor that was previously paired with a foot shock, although the particular brain regions involved in this olfactory paradigm remain to be defined. In another study, lesions of cholinergic inputs from the basal forebrain to the vmPFC results in contextual fear generalization (Knox and Keller, 2016), which the authors suggest is caused by impaired synchronization between the hippocampus and mPFC during fear learning. Finally, NPY is a component of a neuropeptide system that is highly expressed in limbic areas of the brain, where it regulates fear- and anxiety-related behavior (Tasan et al., 2016), while mice lacking NPY or one of its receptors (Y_2_) exhibit heightened generalization to auditory cues (Verma et al., 2012).

### Considerations in the Molecular and Cellular Mechanisms of Fear Generalization

The signaling pathways implicated in the generalization of fear are both diverse and complex, yet they ultimately converge on the excitatory/inhibitory activity of specific cell types within specific brain regions. While much of our current understanding of molecular and cellular mechanisms of fear generalization is derived from the study of proximal time-points, future studies will need to explore how these signaling pathways operate at the level of distinct neural circuits at remote time-points, and as a function of sex. Furthermore, it will be important to investigate how these signaling events impinge on translational, transcriptional, and post-transcriptional processes to modulate the balance between excitatory and inhibitory signaling to produce adaptive or maladaptive behavioral outputs.

## An Integrated Perspective

The generalization of fear is governed by a variety of molecular, cellular, and circuit-level mechanisms that promote the deployment of an optimal defensive response in the face of perceived threat or danger (Maren et al., 2013). Ultimately, the tuning of generalization gradients is a reflection of a complex interplay of multiple internal and external factors, and is shaped by evolutionary imperatives. Although many questions remain as to how this tuning is normally accomplished at a molecular and neural circuit level, and how aberrant tuning might contribute to psychopathology, the evolutionary benefit of generalization itself is clear (e.g., cautious foraging for resources in high-risk environments). Furthermore, the molecular pathways and neural circuits that enable the generalization of experience are governed by positive and negative feedback loops which themselves are dynamic. Indeed, the evolution of complex systems relies on biological networks whose structures are inherently dynamic (Kitano, 2004), and the generalization of fear memories is a prime example of how such networks produce adaptability.

One notable characteristic of the molecular and neural mechanisms described in this review article, whether they promote or inhibit generalization, is that they reflect active processes. As is the case with all aspects of associative memory, including acquisition, consolidation, retrieval, extinction, and forgetting, the generalization of fear is governed by active mechanisms that require significant amounts of energy to regulate highly evolved molecular interactions (Davis and Zhong, 2017). However, it is important to acknowledge the potential contributions of passive mechanisms, despite the fact that these are poorly understood. Given that biological networks are subject to the laws of entropy, it is possible that certain neural circuits are inherently more insulated from the effects of signal degradation than others. Therefore, components of a memory trace may rely on neural circuits that are subject to different rates of decay, leading to imprecise memory (Mensink and Raaijmakers, 1988). Moreover, the entropic decay of elements contained within a memory trace would offer a parsimonious mechanism by which the capacity to generalize may have been shaped by evolution. Furthermore, if synaptic consolidation represents a subroutine of systems consolidation (Dudai et al., 2015), it is likely that even subtle losses in fidelity of the signaling events that govern synaptic plasticity are manifested in higher-order neural functions. Thus, a major focus in the immediate future should be with the identification of whole-brain activity patterns associated with different types of fear generalization (e.g., to contexts and discrete cues) across time.

What can we learn from this discussion of the neurobiology of fear generalization? First, under a certain set of conditions, fear generalization at recent and remote time-points is modulated by the strength of learning (Biedenkapp and Rudy, 2007; Poulos et al., 2016). However, the loss of memory precision driven by forgetting or the inhibitory weakening of the initial memory trace seems to be a continual process. This weakening and loss of precision occurs both in micro-circuits within a brain region (e.g., the central amygdala, dentate gyrus, etc.) as well as in macro-circuits between brain regions (e.g., ventral hippocampus to mPFC; Ciocchi et al., 2010; Cullen et al., 2015). In particular, amygdalar, prefrontal, hippocampal, and thalamic areas appear to be especially important for fear generalization, and a delicate balance between excitatory and inhibitory transmitters, receptors and synapses is critical. Moreover, the neural circuits initially involved in hippocampal-related processes such as pattern separation and pattern completion (Rolls, 2013) may have a different contribution to fear memories and generalization at recent vs. remote time points (Kitamura et al., 2017; Khalaf et al., 2018). Thus, the generalization of fear which occurs at remote timescales likely results from an interaction between the initial associative strength, systems consolidation, and the natural weakening or forgetting of the original memory.

## Future Directions

Understanding the neurobiology of fear generalization is a critical step in the development of novel therapeutic approaches for treating psychiatric disorders such as PTSD. Because fear generalization is conserved across species, animal models are indispensable in the search for causative and potentially exploitable relationships between molecular, cellular, and circuit-level events that influence behavior. With this idea in mind, we emphasize several ideas, both old and new, that should be considered in the design of future studies.

First, given the sexual dimorphisms observed in many psychiatric illnesses, the use of both male and female animals is of paramount importance. Fortunately, at least for several types of fear-related behaviors observed in female C57BL/6 mice, strict monitoring of estrous phase may not be necessary (Meziane et al., 2007; Keiser et al., 2017), and therefore naturally cycling females can be used. In addition, the persistent nature of PTSD and anxiety disorders emphasizes the need to examine remote time-points and other processes such as fear relapse in behavioral experiments (Goode and Maren, 2014; Goode et al., 2018). Also, because psychiatric disorders such as PTSD are highly heritable (Duncan et al., 2018), it will be critical to develop and characterize animal models with genetic alterations at defined loci, whether borne out by GWAS studies or candidate approaches. In this regard, it would be helpful to revisit mouse genetic models that exhibit alterations in fear memory, yet have not been evaluated in remote generalization experiments, given the overlapping circuitry that regulates these processes. Furthermore, because memory traces evolve over time with regard to both their precision and how they are stored in neural circuits, we emphasize the need to evaluate remote changes at the electrophysiological and structural level. For example, optogenetic interrogation of cortical ensembles that are activated during remote fear generalization should help to elucidate the nature of systems consolidation. Finally, cued fear conditioning may be more relevant to short-lasting fear, while contextual conditioning may be more relevant to longer-lasting anxiety states (Davis et al., 2010; Shackman and Fox, 2016; Asok et al., 2018a). Although studies of both types of fear memory have generated a trove of basic neurobiological knowledge, it is likely that contextual models will prove to be especially useful. The breadth and depth of sensory and cognitive experiences associated with a traumatic event in humans suffering from PTSD may be approximated more effectively by a multimodal CS rather than a discrete sensory cue.

In terms of developing new and effective treatments for fear-related disorders, circuit-level approaches such as transcranial magnetic stimulation (Kozel, 2018) and deep brain stimulation (Bina and Langevin, 2018) are promising, although they lack the specificity afforded by pharmacological approaches. The molecular and cellular pathways involved in the processing and storage of fear memories can already be targeted at multiple levels by a vast and extant pharmacopeia with undiscovered capacity to modulate the generalization of fear. However, a given drug target that is expressed throughout the brain can serve distinct functions depending on its subcellular localization in particular brain areas (Engin et al., 2018), making it difficult to modulate specific neural circuits with strictly pharmacological approaches. Therefore, to achieve both molecular and circuit-level specificity, it will be important to capitalize on new technologies that allow cell-type specific targeting of compounds (Nassi et al., 2015; Shields et al., 2017). Well-designed pre-clinical animal studies using targeted delivery of potential therapeutic drugs to examine their effects on fear memory and generalization, across long timescales and as a function of sex, will provide a critical stepping-stone in translating novel compounds from bench to bedside.

## Author Contributions

AA and JR wrote the manuscript. EK provided important conceptual insight and helped prepare the manuscript.

## Conflict of Interest Statement

The authors declare that the research was conducted in the absence of any commercial or financial relationships that could be construed as a potential conflict of interest.

## References

[B1] Abraham A. D. Neve K. A. Lattal K. M. (2016). Effects of D1 receptor knockout on fear and reward learning. Neurobiol. Learn. Mem. 133, 265–273. 10.1016/j.nlm.2016.07.010 27423521 PMC5001556

[B2] Adolphs R. (2013). The biology of fear. Curr. Biol. 23, R79–R93. 10.1016/j.cub.2012.11.055 23347946 PMC3595162

[B3] Andreano J. M. Touroutoglou A. Dickerson B. Barrett L. F. (2018). Hormonal cycles, brain network connectivity, and windows of vulnerability to affective disorder. Trends Neurosci. 41, 660–676. 10.1016/j.tins.2018.08.007 30274602 PMC6481680

[B4] Asok A. Draper A. Hoffman A. F. Schulkin J. Lupica C. R. Rosen J. B. (2018a). Optogenetic silencing of a corticotropin-releasing factor pathway from the central amygdala to the bed nucleus of the stria terminalis disrupts sustained fear. Mol. Psychiatry 23, 914–922. 10.1038/mp.2017.79 28439099 PMC5656568

[B5] Asok A. Leroy F. Rayman J. B. Kandel E. R. (2018b). Molecular mechanisms of the memory trace. Trends Neurosci. [Epub ahead of print]. 10.1016/j.tins.2018.10.005 PMC6312491 30391015

[B6] Baldi E. Lorenzini C. A. Bucherelli C. (2004). Footshock intensity and generalization in contextual and auditory-cued fear conditioning in the rat. Neurobiol. Learn. Mem. 81, 162–166. 10.1016/j.nlm.2004.02.004 15082017

[B7] Balogh S. A. Radcliffe R. A. Logue S. F. Wehner J. M. (2002). Contextual and cued fear conditioning in C57BL/6J and DBA/2J mice: context discrimination and the effects of retention interval. Behav. Neurosci. 116, 947–957. 10.1037//0735-7044.116.6.947 12492293

[B8] Bangasser D. A. Wicks B. (2017). Sex-specific mechanisms for responding to stress. J. Neurosci. Res. 95, 75–82. 10.1002/jnr.23812 27870416 PMC5120612

[B9] Bergado-Acosta J. R. Sangha S. Narayanan R. T. Obata K. Pape H. C. Stork O. (2008). Critical role of the 65-kDa isoform of glutamic acid decarboxylase in consolidation and generalization of Pavlovian fear memory. Learn. Mem. 15, 163–171. 10.1101/lm.705408 18323571 PMC2275658

[B10] Bergstrom H. C. (2016). The neurocircuitry of remote cued fear memory. Neurosci. Biobehav. Rev. 71, 409–417. 10.1016/j.neubiorev.2016.09.028 27693699

[B11] Besnard A. Sahay A. (2016). Adult hippocampal neurogenesis, fear generalization, and stress. Neuropsychopharmacology 41, 24–44. 10.1038/npp.2015.167 26068726 PMC4677119

[B12] Besnard A. Langberg T. Levinson S. Chu D. Vicidomini C. Scobie K. N.. (2018). Targeting kruppel-like factor 9 in excitatory neurons protects against chronic stress-induced impairments in dendritic spines and fear responses. Cell Rep. 23, 3183–3196. 10.1016/j.celrep.2018.05.040 29898391 PMC7453932

[B13] Biedenkapp J. C. Rudy J. W. (2007). Context preexposure prevents forgetting of a contextual fear memory: implication for regional changes in brain activation patterns associated with recent and remote memory tests. Learn. Mem. 14, 200–203. 10.1101/lm.499407 17351145 PMC2519802

[B14] Bina R. W. Langevin J. P. (2018). Closed loop deep brain stimulation for PTSD, addiction, and disorders of affective facial interpretation: review and discussion of potential biomarkers and stimulation paradigms. Front. Neurosci. 12:300. 10.3389/fnins.2018.00300 29780303 PMC5945819

[B16] Blanchard R. J. Blanchard D. C. (1969). Crouching as an index of fear. J. Comp. Physiol. Psychol. 67, 370–375. 10.1037/h0026779 5787388

[B15] Blanchard D. C. Blanchard R. J. (2008). 4 defensive behaviors, fear, and anxiety. Handb. Behav. Neurosci. 17, 63–79. 10.1016/s1569-7339(07)00005-7

[B17] Bouton M. E. Nelson J. B. Rosas J. M. (1999). Stimulus generalization, context change, and forgetting. Psychol. Bull. 125:171. 10.1037/0033-2909.125.2.171 10087934

[B18] Broadbent N. J. Clark R. E. (2013). Remote context fear conditioning remains hippocampus-dependent irrespective of training protocol, training-surgery interval, lesion size, and lesion method. Neurobiol. Learn. Mem. 106, 300–308. 10.1016/j.nlm.2013.08.008 23994542

[B19] Bromberg-Martin E. S. Matsumoto M. Hikosaka O. (2010). Dopamine in motivational control: rewarding, aversive, and alerting. Neuron 68, 815–834. 10.1016/j.neuron.2010.11.022 21144997 PMC3032992

[B20] Brown K. L. Kennard J. A. Sherer D. J. Comalli D. M. Woodruff-Pak D. S. (2011). The context preexposure facilitation effect in mice: a dose-response analysis of pretraining scopolamine administration. Behav. Brain Res. 225, 290–296. 10.1016/j.bbr.2011.07.044 21827794 PMC3179919

[B21] Brunzell D. H. Kim J. J. (2001). Fear conditioning to tone, but not to context, is attenuated by lesions of the insular cortex and posterior extension of the intralaminar complex in rats. Behav. Neurosci. 115, 365–375. 10.1037//0735-7044.115.2.365 11345961

[B22] Bucci D. J. Saddoris M. P. Burwell R. D. (2002). Contextual fear discrimination is impaired by damage to the postrhinal or perirhinal cortex. Behav. Neurosci. 116, 479–488. 10.1037/0735-7044.116.3.479 12049329

[B23] Bueno A. P. A. de Paiva J. P. Q. Corrêa M. D. S. Tiba P. A. Fornari R. V. (2017). Corticosterone administration after a single-trial contextual fear conditioning does not influence the strength and specificity of recent and remote memory in rats. Physiol. Behav. 171, 175–180. 10.1016/j.physbeh.2017.01.011 28082245

[B24] Burghardt N. S. Bauer E. P. (2013). Acute and chronic effects of selective serotonin reuptake inhibitor treatment on fear conditioning: implications for underlying fear circuits. Neuroscience 247, 253–272. 10.1016/j.neuroscience.2013.05.050 23732229

[B25] Canteras N. Swanson L. (1992). Projections of the ventral subiculum to the amygdala, septum, and hypothalamus: a PHAL anterograde tract-tracing study in the rat. J. Comp. Neurol. 324, 180–194. 10.1002/cne.903240204 1430328

[B26] Cenquizca L. A. Swanson L. W. (2007). Spatial organization of direct hippocampal field CA1 axonal projections to the rest of the cerebral cortex. Brain Res. Rev. 56, 1–26. 10.1016/j.brainresrev.2007.05.002 17559940 PMC2171036

[B27] Chua H. C. Chebib M. (2017). GABA_A_ receptors and the diversity in their structure and pharmacology. Adv. Pharmacol. 79, 1–34. 10.1016/bs.apha.2017.03.003 28528665

[B28] Ciocchi S. Herry C. Grenier F. Wolff S. B. Letzkus J. J. Vlachos I.. (2010). Encoding of conditioned fear in central amygdala inhibitory circuits. Nature 468, 277–282. 10.1038/nature09559 21068837

[B29] Ciocchi S. Passecker J. Malagon-Vina H. Mikus N. Klausberger T. (2015). Selective information routing by ventral hippocampal CA1 projection neurons. Science 348, 560–563. 10.1126/science.aaa3245 25931556

[B30] Civelli O. Bunzow J. R. Grandy D. K. (1993). Molecular diversity of the dopamine receptors. Annu. Rev. Pharmacol. Toxicol. 33, 281–307. 10.1146/annurev.pa.33.040193.001433 8494342

[B31] Clark R. E. Sutherland R. J. (2013). The neurobiology of remote memory in the experimental animal. Neurobiol. Learn. Mem. 106, 292–293. 10.1016/j.nlm.2013.11.001 24295497

[B32] Cohen H. Zohar J. (2004). An animal model of posttraumatic stress disorder: the use of cut-off behavioral criteria. Ann. N Y Acad. Sci. 1032, 167–178. 10.1196/annals.1314.014 15677404

[B33] Cohen H. Zohar J. Matar M. (2003). The relevance of differential response to trauma in an animal model of posttraumatic stress disorder. Biol. Psychiatry 53, 463–473. 10.1016/s0006-3223(02)01909-1 12644351

[B34] Cohen H. Zohar J. Matar M. A. Zeev K. Loewenthal U. Richter-Levin G. (2004). Setting apart the affected: the use of behavioral criteria in animal models of post traumatic stress disorder. Neuropsychopharmacology 29, 1962–1970. 10.1038/sj.npp.1300523 15257304

[B35] Cooper W. E. Blumstein D. T. (2015). Escaping From Predators: An Integrative View of Escape Decisions. Cambridge: Cambridge University Press.

[B36] Cortese B. M. Leslie K. Uhde T. W. (2015). Differential odor sensitivity in PTSD: implications for treatment and future research. J. Affect. Disord. 179, 23–30. 10.1016/j.jad.2015.03.026 25845746 PMC4437877

[B37] Cravens C. J. Vargas-Pinto N. Christian K. M. Nakazawa K. (2006). CA3 NMDA receptors are crucial for rapid and automatic representation of context memory. Eur. J. Neurosci. 24, 1771–1780. 10.1111/j.1460-9568.2006.05044.x 17004940

[B38] Cullen P. K. Dulka B. N. Ortiz S. Riccio D. C. Jasnow A. M. (2014). GABA-mediated presynaptic inhibition is required for precision of long-term memory. Learn. Mem. 21, 180–184. 10.1101/lm.032961.113 24634352 PMC3966537

[B39] Cullen P. K. Gilman T. L. Winiecki P. Riccio D. C. Jasnow A. M. (2015). Activity of the anterior cingulate cortex and ventral hippocampus underlie increases in contextual fear generalization. Neurobiol. Learn. Mem. 124, 19–27. 10.1016/j.nlm.2015.07.001 26165137

[B40] Darwin C. (1888). The Descent of Man and Selection in Relation to Sex. London: Murray.

[B41] Davis M. (2006). Neural systems involved in fear and anxiety measured with fear-potentiated startle. Am. Psychol. 61, 741–756. 10.1037/0003-066X.61.8.741 17115806

[B42] Davis M. Walker D. L. Miles L. Grillon C. (2010). Phasic vs sustained fear in rats and humans: role of the extended amygdala in fear vs anxiety. Neuropsychopharmacology 35, 105–135. 10.1038/npp.2009.109 19693004 PMC2795099

[B43] Davis R. L. Zhong Y. (2017). The biology of forgetting-A perspective. Neuron 95, 490–503. 10.1016/j.neuron.2017.05.039 28772119 PMC5657245

[B44] Day H. L. L. Reed M. M. Stevenson C. W. (2016). Sex differences in discriminating between cues predicting threat and safety. Neurobiol. Learn. Mem. 133, 196–203. 10.1016/j.nlm.2016.07.014 27423522 PMC4993817

[B45] De Bundel D. Zussy C. Espallergues J. Gerfen C. R. Girault J. A. Valjent E. (2016). Dopamine D2 receptors gate generalization of conditioned threat responses through mTORC1 signaling in the extended amygdala. Mol. Psychiatry 21, 1545–1553. 10.1038/mp.2015.210 26782052 PMC5101541

[B46] de Kloet E. R. Joels M. Holsboer F. (2005). Stress and the brain: from adaptation to disease. Nat. Rev. Neurosci. 6, 463–475. 10.1038/nrn1683 15891777

[B47] Desmedt A. Marighetto A. Garcia R. Jaffard R. (2003). The effects of ibotenic hippocampal lesions on discriminative fear conditioning to context in mice: impairment or facilitation depending on the associative value of a phasic explicit cue. Eur. J. Neurosci. 17, 1953–1963. 10.1046/j.1460-9568.2003.02615.x 12752795

[B48] Dong H.-W. Petrovich G. D. Swanson L. W. (2001). Topography of projections from amygdala to bed nuclei of the stria terminalis. Brain Res. Rev. 38, 192–246. 10.1016/s0165-0173(01)00079-0 11750933

[B49] Donley M. P. Schulkin J. Rosen J. B. (2005). Glucocorticoid receptor antagonism in the basolateral amygdala and ventral hippocampus interferes with long-term memory of contextual fear. Behav. Brain Res. 164, 197–205. 10.1016/j.bbr.2005.06.020 16107281

[B50] Dudai Y. (2004). The neurobiology of consolidations, or, how stable is the engram? Annu. Rev. Psychol. 55, 51–86. 10.1146/annurev.psych.55.090902.142050 14744210

[B51] Dudai Y. Karni A. Born J. (2015). The consolidation and transformation of memory. Neuron 88, 20–32. 10.1016/j.neuron.2015.09.004 26447570

[B52] Duncan L. E. Ratanatharathorn A. Aiello A. E. Almli L. M. Amstadter A. B. Ashley-Koch A. E.. (2018). Largest GWAS of PTSD (N=20 070) yields genetic overlap with schizophrenia and sex differences in heritability. Mol. Psychiatry 23, 666–673. 10.1038/mp.2017.77 28439101 PMC5696105

[B53] Dunsmoor J. E. Paz R. (2015). Fear generalization and anxiety: behavioral and neural mechanisms. Biol. Psychiatry 78, 336–343. 10.1016/j.biopsych.2015.04.010 25981173

[B54] Dunsmoor J. E. Prince S. E. Murty V. P. Kragel P. A. LaBar K. S. (2011). Neurobehavioral mechanisms of human fear generalization. Neuroimage 55, 1878–1888. 10.1016/j.neuroimage.2011.01.041 21256233 PMC3062636

[B55] Duvarci S. Bauer E. P. Paré D. (2009). The bed nucleus of the stria terminalis mediates inter-individual variations in anxiety and fear. J. Neurosci. 29, 10357–10361. 10.1523/JNEUROSCI.2119-09.2009 19692610 PMC2741739

[B56] Elliott N. D. Richardson R. (2018). The effects of early life stress on context fear generalization in adult rats. Behav. Neurosci. [Epub ahead of print]. 10.1037/bne0000289 30489134

[B57] Elzinga B. M. Bremner J. D. (2002). Are the neural substrates of memory the final common pathway in posttraumatic stress disorder (PTSD)? J. Affect. Disord. 70, 1–17. 10.1016/s0165-0327(01)00351-2 12113915 PMC5580811

[B58] Engin E. Benham R. S. Rudolph U. (2018). An emerging circuit pharmacology of GABA_A_ receptors. Trends Pharmacol. Sci. 39, 710–732. 10.1016/j.tips.2018.04.003 29903580 PMC6056379

[B59] Fanselow M. S. (1994). Neural organization of the defensive behavior system responsible for fear. Psychon. Bull. Rev. 1, 429–438. 10.3758/BF03210947 24203551

[B60] Fanselow M. S. Dong H.-W. (2010). Are the dorsal and ventral hippocampus functionally distinct structures? Neuron 65, 7–19. 10.1016/j.neuron.2009.11.031 20152109 PMC2822727

[B61] Fendt M. Fanselow M. S. (1999). The neuroanatomical and neurochemical basis of conditioned fear. Neurosci. Biobehav. Rev. 23, 743–760. 10.1016/s0149-7634(99)00016-0 10392663

[B62] Ferrara N. C. Cullen P. K. Pullins S. P. Rotondo E. K. Helmstetter F. J. (2017). Input from the medial geniculate nucleus modulates amygdala encoding of fear memory discrimination. Learn. Mem. 24, 414–421. 10.1101/lm.044131.116 28814467 PMC5580525

[B63] Frangaj A. Fan Q. R. (2018). Structural biology of GABAB receptor. Neuropharmacology 136, 68–79. 10.1016/j.neuropharm.2017.10.011 29031577 PMC5897222

[B64] Frankland P. W. Bontempi B. Talton L. E. Kaczmarek L. Silva A. J. (2004). The involvement of the anterior cingulate cortex in remote contextual fear memory. Science 304, 881–883. 10.1126/science.1094804 15131309

[B65] Froemke R. C. (2015). Plasticity of cortical excitatory-inhibitory balance. Annu. Rev. Neurosci. 38, 195–219. 10.1146/annurev-neuro-071714-034002 25897875 PMC4652600

[B66] Gazarini L. Stern C. A. Carobrez A. P. Bertoglio L. J. (2013). Enhanced noradrenergic activity potentiates fear memory consolidation and reconsolidation by differentially recruiting α1- and β-adrenergic receptors. Learn. Mem. 20, 210–219. 10.1101/lm.030007.112 23512937

[B68] Goode T. D. Jin J. Maren S. (2018). “Neural circuits for fear relapse,” in Neurobiology of Abnormal Emotion and Motivated Behaviors, eds Sangha S. Foti D. (London: Elsevier), 182–202.

[B67] Goode T. D. Maren S. (2014). Animal models of fear relapse. ILAR J. 55, 246–258. 10.1093/ilar/ilu008 25225304 PMC4197897

[B69] Goosens K. A. Maren S. (2001). Contextual and auditory fear conditioning are mediated by the lateral, basal, and central amygdaloid nuclei in rats. Learn. Mem. 8, 148–155. 10.1101/lm.37601 11390634 PMC311374

[B70] Gross C. T. Canteras N. S. (2012). The many paths to fear. Nat. Rev. Neurosci. 13, 651–658. 10.1038/nrn3301 22850830

[B71] Grosso A. Santoni G. Manassero E. Renna A. Sacchetti B. (2018). A neuronal basis for fear discrimination in the lateral amygdala. Nat. Commun. 9:1214. 10.1038/s41467-018-03682-2 29572443 PMC5865209

[B72] Gruene T. M. Flick K. Stefano A. Shea S. D. Shansky R. M. (2015). Sexually divergent expression of active and passive conditioned fear responses in rats. Elife 4:e11352. 10.7554/eLife.11352 26568307 PMC4709260

[B73] Guttman N. Kalish H. I. (1956). Discriminability and stimulus generalization. J. Exp. Psychol. 51, 79–88. 10.1037/h0046219 13286444

[B74] Han J. H. Yiu A. P. Cole C. J. Hsiang H. L. Neve R. L. Josselyn S. A. (2008). Increasing CREB in the auditory thalamus enhances memory and generalization of auditory conditioned fear. Learn. Mem. 15, 443–453. 10.1101/lm.993608 18519545 PMC2414255

[B75] Holland P. C. Bouton M. E. (1999). Hippocampus and context in classical conditioning. Curr. Opin. Neurobiol. 9, 195–202. 10.1016/s0959-4388(99)80027-0 10322181

[B76] Homberg J. R. (2012). Serotonergic modulation of conditioned fear. Scientifica 2012:821549. 10.6064/2012/821549 24278743 PMC3820492

[B77] Huckleberry K. A. Ferguson L. B. Drew M. R. (2016). Behavioral mechanisms of context fear generalization in mice. Learn. Mem. 23, 703–709. 10.1101/lm.042374.116 27918275 PMC5110986

[B78] Hull C. L. (1943). Principles of Behavior: An Introduction to Behavior Theory. New York, NY: Appleton-Century-Crofts.

[B79] Ikegami M. Uemura T. Kishioka A. Sakimura K. Mishina M. (2014). Striatal dopamine D1 receptor is essential for contextual fear conditioning. Sci. Rep. 4:3976. 10.1038/srep03976 24496082 PMC3913917

[B80] Ishikawa R. Fukushima H. Frankland P. W. Kida S. (2016). Hippocampal neurogenesis enhancers promote forgetting of remote fear memory after hippocampal reactivation by retrieval. Elife 5:e17464. 10.7554/eLife.17464 27669409 PMC5036964

[B81] Jasnow A. M. Cullen P. K. Riccio D. C. (2012). Remembering another aspect of forgetting. Front. Psychol. 3:175. 10.3389/fpsyg.2012.00175 22675315 PMC3365651

[B82] Jasnow A. M. Lynch J. F. III. Gilman T. L. Riccio D. C. (2017). Perspectives on fear generalization and its implications for emotional disorders. J. Neurosci. Res. 95, 821–835. 10.1002/jnr.23837 27448175

[B83] Jimenez J. C. Su K. Goldberg A. R. Luna V. M. Biane J. S. Ordek G.. (2018). Anxiety cells in a hippocampal-hypothalamic circuit. Neuron 97, 670.e6–683.e6. 10.1016/j.neuron.2018.01.016 29397273 PMC5877404

[B84] Johnson E. O. Kamilaris T. C. Chrousos G. P. Gold P. W. Reviews B. (1992). Mechanisms of stress: a dynamic overview of hormonal and behavioral homeostasis. Neurosci. Biobehav. Rev. 16, 115–130. 10.1016/s0149-7634(05)80175-7 1630726

[B85] Jones G. L. Soden M. E. Knakal C. R. Lee H. Chung A. S. Merriam E. B.. (2015). A genetic link between discriminative fear coding by the lateral amygdala, dopamine, and fear generalization. Elife 4:e08969. 10.7554/eLife.08969 26402461 PMC4621744

[B86] Kaczkurkin A. N. Burton P. C. Chazin S. M. Manbeck A. B. Espensen-Sturges T. Cooper S. E.. (2016). Neural substrates of overgeneralized conditioned fear in PTSD. Am. J. Psychiatry 174, 125–134. 10.1176/appi.ajp.2016.15121549 27794690 PMC7269602

[B87] Kahnt T. Tobler P. N. (2016). Dopamine regulates stimulus generalization in the human hippocampus. Elife 5:e12678. 10.7554/elife.12678 26830462 PMC4755747

[B88] Kano M. Ohno-Shosaku T. Hashimotodani Y. Uchigashima M. Watanabe M. (2009). Endocannabinoid-mediated control of synaptic transmission. Physiol. Rev. 89, 309–380. 10.1152/physrev.00019.2008 19126760

[B89] Kaouane N. Porte Y. Vallée M. Brayda-Bruno L. Mons N. Calandreau L.. (2012). Glucocorticoids can induce PTSD-like memory impairments in mice. Science 335, 1510–1513. 10.1126/science.1207615 22362879

[B90] Keiser A. A. Turnbull L. M. Darian M. A. Feldman D. E. Song I. Tronson N. C. (2017). Sex differences in context fear generalization and recruitment of hippocampus and amygdala during retrieval. Neuropsychopharmacology 42, 397–407. 10.1038/npp.2016.174 27577601 PMC5399239

[B91] Kelley D. B. (1988). Sexually dimorphic behaviors. Annu. Rev. Neurosci. 11, 225–251. 10.1146/annurev.neuro.11.1.225 3284441

[B92] Kessler R. C. Chiu W. T. Demler O. Merikangas K. R. Walters E. E. (2005). Prevalence, severity, and comorbidity of 12-month DSM-IV disorders in the national comorbidity survey replication. Arch. Gen. Psychiatry 62, 617–627. 10.1001/archpsyc.62.6.617 15939839 PMC2847357

[B93] Kessler R. C. Petukhova M. Sampson N. A. Zaslavsky A. M. Wittchen H.-U. (2012). Twelve-month and lifetime prevalence and lifetime morbid risk of anxiety and mood disorders in the United States. Int. J. Methods Psychiatr. Res. 21, 169–184. 10.1002/mpr.1359 22865617 PMC4005415

[B94] Khalaf O. Resch S. Dixsaut L. Gorden V. Glauser L. Gräff J. (2018). Reactivation of recall-induced neurons contributes to remote fear memory attenuation. Science 360, 1239–1242. 10.1126/science.aas9875 29903974

[B95] Kitamura T. Ogawa S. K. Roy D. S. Okuyama T. Morrissey M. D. Smith L. M.. (2017). Engrams and circuits crucial for systems consolidation of a memory. Science 356, 73–78. 10.1126/science.aam6808 28386011 PMC5493329

[B96] Kitano H. (2004). Biological robustness. Nat. Rev. Genet. 5, 826–837. 10.1038/nrg1471 15520792

[B97] Klemenhagen K. C. Gordon J. A. David D. J. Hen R. Gross C. T. (2006). Increased fear response to contextual cues in mice lacking the 5-HT1A receptor. Neuropsychopharmacology 31, 101–111. 10.1038/sj.npp.1300774 15920501

[B98] Knox D. Keller S. M. (2016). Cholinergic neuronal lesions in the medial septum and vertical limb of the diagonal bands of Broca induce contextual fear memory generalization and impair acquisition of fear extinction. Hippocampus 26, 718–726. 10.1002/hipo.22553 26606423 PMC5496651

[B99] Koch C. Leinweber B. Drengberg B. Blaum C. Oster H. (2017). Interaction between circadian rhythms and stress. Neurobiol. Stress 6, 57–67. 10.1016/j.ynstr.2016.09.001 28229109 PMC5314421

[B100] Kozel F. A. (2018). Clinical repetitive transcranial magnetic stimulation for posttraumatic stress disorder, generalized anxiety disorder, and bipolar disorder. Psychiatr. Clin. North Am. 41, 433–446. 10.1016/j.psc.2018.04.007 30098656

[B101] Krasne F. B. Cushman J. D. Fanselow M. S. (2015). A Bayesian context fear learning algorithm/automaton. Front. Behav. Neurosci. 9:112. 10.3389/fnbeh.2015.00112 26074792 PMC4445248

[B102] Lanuza E. Moncho-Bogani J. LeDoux J. E. (2008). Unconditioned stimulus pathways to the amygdala: effects of lesions of the posterior intralaminar thalamus on foot-shock-induced c-Fos expression in the subdivisions of the lateral amygdala. Neuroscience 155, 959–968. 10.1016/j.neuroscience.2008.06.028 18620025 PMC2587439

[B103] Lanuza E. Nader K. Ledoux J. E. (2004). Unconditioned stimulus pathways to the amygdala: effects of posterior thalamic and cortical lesions on fear conditioning. Neuroscience 125, 305–315. 10.1016/j.neuroscience.2003.12.034 15062974

[B104] Lashley K. S. Wade M. (1946). The Pavlovian theory of generalization. Psychol. Rev. 53, 72–87. 10.1037/h0059999 21023320

[B105] LeDoux J. (2012). Rethinking the emotional brain. Neuron 73, 653–676. 10.1016/j.neuron.2012.02.004 22365542 PMC3625946

[B106] Lee I. Lee C. H. (2013). Contextual behavior and neural circuits. Front. Neural Circuits 7:84. 10.3389/fncir.2013.00084 23675321 PMC3650478

[B107] Lehmann H. Lacanilao S. Sutherland R. J. (2007). Complete or partial hippocampal damage produces equivalent retrograde amnesia for remote contextual fear memories. Eur. J. Neurosci. 25, 1278–1286. 10.1111/j.1460-9568.2007.05374.x 17355254

[B108] Linke R. Braune G. Schwegler H. (2000). Differential projection of the posterior paralaminar thalamic nuclei to the amygdaloid complex in the rat. Exp. Brain Res. 134, 520–532. 10.1007/s002210000475 11081834

[B109] Lissek S. Bradford D. E. Alvarez R. P. Burton P. Espensen-Sturges T. Reynolds R. C.. (2013). Neural substrates of classically conditioned fear-generalization in humans: a parametric fMRI study. Soc. Cogn. Affect. Neurosci. 9, 1134–1142. 10.1093/scan/nst096 23748500 PMC4127021

[B110] Lissek S. Rabin S. Heller R. E. Lukenbaugh D. Geraci M. Pine D. S.. (2010). Overgeneralization of conditioned fear as a pathogenic marker of panic disorder. Am. J. Psychiatry 167, 47–55. 10.1176/appi.ajp.2009.09030410 19917595 PMC2806514

[B111] Lynch J. F. III. Cullen P. K. Jasnow A. M. Riccio D. C. (2013). Sex differences in the generalization of fear as a function of retention intervals. Learn. Mem. 20, 628–632. 10.1101/lm.032011.113 24131793

[B112] Lynch J. F. III. Vanderhoof T. Winiecki P. Latsko M. S. Riccio D. C. Jasnow A. M. (2016a). Aromatized testosterone attenuates contextual generalization of fear in male rats. Horm. Behav. 84, 127–135. 10.1016/j.yhbeh.2016.06.007 27368147

[B113] Lynch J. F. III. Winiecki P. Vanderhoof T. Riccio D. C. Jasnow A. M. (2016b). Hippocampal cytosolic estrogen receptors regulate fear generalization in females. Neurobiol. Learn. Mem. 130, 83–92. 10.1016/j.nlm.2016.01.010 26851128

[B114] Malin E. L. McGaugh J. L. (2006). Differential involvement of the hippocampus, anterior cingulate cortex, and basolateral amygdala in memory for context and footshock. Proc. Natl. Acad. Sci. U S A 103, 1959–1963. 10.1073/pnas.0510890103 16446423 PMC1413673

[B115] Maren S. (2001). Neurobiology of Pavlovian fear conditioning. Annu. Rev. Neurosci. 24, 897–931. 10.1146/annurev.neuro.24.1.897 11520922

[B116] Maren S. Aharonov G. Fanselow M. S. (1997). Neurotoxic lesions of the dorsal hippocampus and Pavlovian fear conditioning in rats. Behav. Brain Res. 88, 261–274. 10.1016/s0166-4328(97)00088-0 9404635

[B117] Maren S. Fanselow M. S. (1995). Synaptic plasticity in the basolateral amygdala induced by hippocampal formation stimulation *in vivo*. J. Neurosci. 15, 7548–7564. 10.1523/JNEUROSCI.15-11-07548.1995 7472506 PMC6578043

[B118] Maren S. Phan K. L. Liberzon I. (2013). The contextual brain: implications for fear conditioning, extinction and psychopathology. Nat. Rev. Neurosci. 14, 417–428. 10.1038/nrn3492 23635870 PMC5072129

[B119] Matsuo N. (2015). Irreplaceability of neuronal ensembles after memory allocation. Cell Rep. 11, 351–357. 10.1016/j.celrep.2015.03.042 25900079

[B120] McAllister W. R. McAllister D. E. (1963). Increase over time in the stimulus generalization of acquired fear. J. Exp. Psychol. 65, 576–582. 10.1037/h0046583

[B121] McEwen B. S. (1998). Stress, adaptation, and disease: allostasis and allostatic load. Ann. N Y Acad. Sci. 840, 33–44. 10.1111/j.1749-6632.1998.tb09546.x 9629234

[B122] McGaugh J. L. (2013). Making lasting memories: remembering the significant. Proc. Natl. Acad. Sci. U S A 110, 10402–10407. 10.1073/pnas.1301209110 23754441 PMC3690616

[B124] McHugh T. J. Jones M. W. Quinn J. J. Balthasar N. Coppari R. Elmquist J. K.. (2007). Dentate gyrus NMDA receptors mediate rapid pattern separation in the hippocampal network. Science 317, 94–99. 10.1126/science.1140263 17556551

[B123] McHugh T. J. Tonegawa S. (2007). Spatial exploration is required for the formation of contextual fear memory. Behav. Neurosci. 121, 335–339. 10.1037/0735-7044.121.2.335 17469922

[B125] McIntyre C. K. McGaugh J. L. Williams C. L. (2012). Interacting brain systems modulate memory consolidation. Neurosci. Biobehav. Rev. 36, 1750–1762. 10.1016/j.neubiorev.2011.11.001 22085800 PMC3315607

[B126] McReynolds J. R. Donowho K. Abdi A. McGaugh J. L. Roozendaal B. McIntyre C. K. (2010). Memory-enhancing corticosterone treatment increases amygdala norepinephrine and Arc protein expression in hippocampal synaptic fractions. Neurobiol. Learn. Mem. 93, 312–321. 10.1016/j.nlm.2009.11.005 19932757 PMC5639692

[B127] Meir Drexler S. Wolf O. T. (2017). The role of glucocorticoids in emotional memory reconsolidation. Neurobiol. Learn. Mem. 142, 126–134. 10.1016/j.nlm.2016.11.008 27871996

[B128] Mensink G.-J. Raaijmakers J. G. (1988). A model for interference and forgetting. Psychol. Rev. 95, 434–455. 10.1037//0033-295x.95.4.434

[B129] Meziane H. Ouagazzal A. M. Aubert L. Wietrzych M. Krezel W. (2007). Estrous cycle effects on behavior of C57BL/6J and BALB/cByJ female mice: implications for phenotyping strategies. Genes Brain Behav. 6, 192–200. 10.1111/j.1601-183x.2006.00249.x 16827921

[B130] Migues P. V. Liu L. Archbold G. E. Einarsson E. Ö. Wong J. Bonasia K.. (2016). Blocking synaptic removal of GluA2-containing AMPA receptors prevents the natural forgetting of long-term memories. J. Neurosci. 36, 3481–3494. 10.1523/JNEUROSCI.3333-15.2016 27013677 PMC6601735

[B131] Miller R. R. Polack C. W. (2018). Sources of maladaptive behavior in ‘normal’ organisms. Behav. Processes 154, 4–12. 10.1016/j.beproc.2017.12.017 29274378 PMC6013324

[B132] Mongillo G. Rumpel S. Loewenstein Y. (2018). Inhibitory connectivity defines the realm of excitatory plasticity. Nat. Neurosci. 21, 1463–1470. 10.1038/s41593-018-0226-x 30224809

[B133] Moscovitch M. Nadel L. (1999). Multiple-trace theory and semantic dementia: response to K.S. Graham (1999). Trends Cogn. Sci. 3, 87–89. 10.1016/s1364-6613(99)01290-5 10322459

[B134] Moscovitch M. Rosenbaum R. S. Gilboa A. Addis D. R. Westmacott R. Grady C.. (2005). Functional neuroanatomy of remote episodic, semantic and spatial memory: a unified account based on multiple trace theory. J. Anat. 207, 35–66. 10.1111/j.1469-7580.2005.00421.x 16011544 PMC1571502

[B135] Murawski N. J. Asok A. (2017). Understanding the contributions of visual stimuli to contextual fear conditioning: a proof-of-concept study using LCD screens. Neurosci. Lett. 637, 80–84. 10.1016/j.neulet.2016.11.046 27888041 PMC6836723

[B136] Nabavi S. Fox R. Proulx C. D. Lin J. Y. Tsien R. Y. Malinow R. (2014). Engineering a memory with LTD and LTP. Nature 511, 348–352. 10.1038/nature13294 24896183 PMC4210354

[B137] Nakashiba T. Cushman J. D. Pelkey K. A. Renaudineau S. Buhl D. L. McHugh T. J.. (2012). Young dentate granule cells mediate pattern separation, whereas old granule cells facilitate pattern completion. Cell 149, 188–201. 10.1016/j.cell.2012.01.046 22365813 PMC3319279

[B138] Nassi J. J. Cepko C. L. Born R. T. Beier K. T. (2015). Neuroanatomy goes viral! Front. Neuroanat. 9:80. 10.3389/fnana.2015.00080 26190977 PMC4486834

[B139] O’Reilly R. C. Rudy J. W. (2001). Conjunctive representations in learning and memory: principles of cortical and hippocampal function. Psychol. Rev. 108, 311–345. 10.1037/0033-295x.108.2.311 11381832

[B140] Ortiz O. Delgado-Garcia J. M. Espadas I. Bahí A. Trullas R. Dreyer J. L.. (2010). Associative learning and CA3-CA1 synaptic plasticity are impaired in D_1_R null, *Drd1a^−/−^* mice and in hippocampal siRNA silenced *Drd1a* mice. J. Neurosci. 30, 12288–12300. 10.1523/JNEUROSCI.2655-10.2010 20844125 PMC6633447

[B141] Paré D. Quirk G. J. Ledoux J. E. (2004). New vistas on amygdala networks in conditioned fear. J. Neurophysiol. 92, 1–9. 10.1152/jn.00153.2004 15212433

[B142] Pavesi E. Heldt S. A. Fletcher M. L. (2013). Neuronal nitric-oxide synthase deficiency impairs the long-term memory of olfactory fear learning and increases odor generalization. Learn. Mem. 20, 482–490. 10.1101/lm.031450.113 23955171

[B143] Pavlov I. P. (1927). Conditional Reflexes: An Investigation of the Physiological Activity of the Cerebral Cortex. Oxford, England: Oxford University Press.

[B144] Pearce J. M. (1987). A model for stimulus generalization in Pavlovian conditioning. Psychol. Rev. 94, 61–73. 10.1037/0033-295x.94.1.61 3823305

[B145] Pezze M. A. Feldon J. (2004). Mesolimbic dopaminergic pathways in fear conditioning. Prog. Neurobiol. 74, 301–320. 10.1016/j.pneurobio.2004.09.004 15582224

[B146] Pitkänen A. Pikkarainen M. Nurminen N. Ylinen A. (2000). Reciprocal connections between the amygdala and the hippocampal formation, perirhinal cortex, and postrhinal cortex in rat: a review. Ann. N Y Acad. Sci. 911, 369–391. 10.1111/j.1749-6632.2000.tb06738.x 10911886

[B147] Pollack G. A. Bezek J. L. Lee S. H. Scarlata M. J. Weingast L. T. Bergstrom H. C. (2018). Cued fear memory generalization increases over time. Learn. Mem. 25, 298–308. 10.1101/lm.047555.118 29907637 PMC6004064

[B148] Poulos A. M. Mehta N. Lu B. Amir D. Livingston B. Santarelli A.. (2016). Conditioning-and time-dependent increases in context fear and generalization. Learn. Mem. 23, 379–385. 10.1101/lm.041400.115 27317198 PMC4918784

[B149] Radulovic J. Kammermeier J. Spiess J. (1998). Generalization of fear responses in C57BL/6N mice subjected to one-trial foreground contextual fear conditioning. Behav. Brain Res. 95, 179–189. 10.1016/s0166-4328(98)00039-4 9806438

[B150] Rajbhandari A. K. Zhu R. Adling C. Fanselow M. S. Waschek J. A. (2016). Graded fear generalization enhances the level of cfos-positive neurons specifically in the basolateral amygdala. J. Neurosci. Res. 94, 1393–1399. 10.1002/jnr.23947 27661774 PMC5069180

[B151] Ramanathan K. R. Ressler R. L. Jin J. Maren S. (2018). Nucleus reuniens is required for encoding and retrieving precise, hippocampal-dependent contextual fear memories in rats. J. Neurosci. 38, 9925–9933. 10.1523/JNEUROSCI.1429-18.2018 30282726 PMC6234294

[B152] Rekkas P. V. Constable R. T. (2005). Evidence that autobiographic memory retrieval does not become independent of the hippocampus: an fMRI study contrasting very recent with remote events. J. Cogn. Neurosci. 17, 1950–1961. 10.1162/089892905775008652 16475281

[B153] Rescorla R. A. (1976). Stimulus generalization: some predictions from a model of Pavlovian conditioning. J. Exp. Psychol. Anim. Behav. Processes 2, 88–96. 10.1037/0097-7403.2.1.88 1249526

[B154] Rescorla R. A. Wagner A. R. (1972). “A theory of Pavlovian conditioning: variations in the effectiveness of reinforcement and nonreinforcement,” in Classical Conditioning II: Current Research and Theory, (Vol. 2) eds Black A. H. Prokasy W. F. (New York, NY: Appleton-Century-Crofts), 64–99.

[B155] Riccio D. C. Ackil J. K. Burch-Vernon A. (1992). Forgetting of stimulus attributes: methodological implications for assessing associative phenomena. Psychol. Bull. 112, 433–445. 10.1037/0033-2909.112.3.433 1438637

[B156] Richards B. A. Frankland P. W. (2017). The persistence and transience of memory. Neuron 94, 1071–1084. 10.1016/j.neuron.2017.04.037 28641107

[B157] Richter-Levin G. Stork O. Schmidt M. V. (2018). Animal models of PTSD: a challenge to be met. Mol. Psychiatry [Epub ahead of print]. 10.1038/s41380-018-0272-5 PMC6756084 30816289

[B158] Rodrigues S. M. LeDoux J. E. Sapolsky R. M. (2009). The influence of stress hormones on fear circuitry. Annu. Rev. Neurosci. 32, 289–313. 10.1146/annurev.neuro.051508.135620 19400714

[B159] Rohrbaugh M. Riccio D. C. (1968). Stimulus generalization of learned fear in infant and adult rats. J. Comp. Physiol. Psychol. 66, 530–533. 10.1037/h0026366 5722071

[B161] Rolls E. (2013). The mechanisms for pattern completion and pattern separation in the hippocampus. Front. Syst. Neurosci. 7:74. 10.3389/fnsys.2013.00074 24198767 PMC3812781

[B160] Rolls A. Makam M. Kroeger D. Colas D. D. de Lecea L. Heller H. C. (2013). Sleep to forget: interference of fear memories during sleep. Mol. Psychiatry 18, 1166–1170. 10.1038/mp.2013.121 24081009 PMC5036945

[B162] Roozendaal B. McEwen B. S. Chattarji S. (2009). Stress, memory and the amygdala. Nat. Rev. Neurosci. 10, 423–433. 10.1038/nrn2651 19469026

[B163] Roy D. S. Kitamura T. Okuyama T. Ogawa S. K. Sun C. Obata Y.. (2017). Distinct neural circuits for the formation and retrieval of episodic memories. Cell 170, 1000–1012. 10.1016/j.cell.2017.07.013 28823555 PMC5586038

[B164] Rozeske R. R. Valerio S. Chaudun F. Herry C. (2015). Prefrontal neuronal circuits of contextual fear conditioning. Genes Brain Behav. 14, 22–36. 10.1111/gbb.12181 25287656

[B166] Rudy J. W. (2009). Context representations, context functions, and the parahippocampal—hippocampal system. Learn. Mem. 16, 573–585. 10.1101/lm.1494409 19794181 PMC2769166

[B168] Rudy J. W. Biedenkapp J. C. O’Reilly R. C. (2005). Prefrontal cortex and the organization of recent and remote memories: an alternative view. Learn. Mem. 12, 445–446. 10.1101/lm.97905 16166399

[B165] Rudy J. W. Huff N. Matus-Amat P. (2004). Understanding contextual fear conditioning: insights from a two-process model. Neurosci. Biobehav. Rev. 28, 675–685. 10.1016/j.neubiorev.2004.09.004 15555677

[B167] Rudy J. W. O’Reilly R. C. (1999). Contextual fear conditioning, conjunctive representations, pattern completion, and the hippocampus. Behav. Neurosci. 113, 867–880. 10.1037/0735-7044.113.5.867 10571471

[B169] Ruediger S. Vittori C. Bednarek E. Genoud C. Strata P. Sacchetti B.. (2011). Learning-related feedforward inhibitory connectivity growth required for memory precision. Nature 473, 514–518. 10.1038/nature09946 21532590

[B170] Sahay A. Scobie K. N. Hill A. S. O’Carroll C. M. Kheirbek M. A. Burghardt N. S.. (2011). Increasing adult hippocampal neurogenesis is sufficient to improve pattern separation. Nature 472, 466–470. 10.1038/nature09817 21460835 PMC3084370

[B171] Sakurai T. (2007). The neural circuit of orexin (hypocretin): maintaining sleep and wakefulness. Nat. Rev. Neurosci. 8, 171–181. 10.1038/nrn2092 17299454

[B172] Sanford C. A. Soden M. E. Baird M. A. Miller S. M. Schulkin J. Palmiter R. D.. (2017). A central amygdala CRF circuit facilitates learning about weak threats. Neuron 93, 164–178. 10.1016/j.neuron.2016.11.034 28017470 PMC5217711

[B173] Sangha S. Narayanan R. T. Bergado-Acosta J. R. Stork O. Seidenbecher T. Pape H. C. (2009). Deficiency of the 65 kDa isoform of glutamic acid decarboxylase impairs extinction of cued but not contextual fear memory. J. Neurosci. 29, 15713–15720. 10.1523/JNEUROSCI.2620-09.2009 20016086 PMC6666166

[B174] Sarinana J. Kitamura T. Künzler P. Sultzman L. Tonegawa S. (2014). Differential roles of the dopamine 1-class receptors, D1R and D5R, in hippocampal dependent memory. Proc. Natl. Acad. Sci. U S A 111, 8245–8250. 10.1073/pnas.1407395111 24843151 PMC4050601

[B175] Schultz W. Dayan P. Montague P. R. (1997). A neural substrate of prediction and reward. Science 275, 1593–1599. 10.1126/science.275.5306.1593 9054347

[B176] Schwabe L. Joëls M. Roozendaal B. Wolf O. T. Oitzl M. S. (2012). Stress effects on memory: an update and integration. Neurosci. Biobehav. Rev. 36, 1740–1749. 10.1016/j.neubiorev.2011.07.002 21771612

[B177] Sekeres M. J. Moscovitch M. Winocur G. (2017). “Mechanisms of memory consolidation and transformation,” in Studies in Neuroscience, Psychology and Behavioral Economics. Cognitive Neuroscience of Memory Consolidation, eds Axmacher N. Rasch B. (Cham, Switzerland: Springer International Publishing), 17–44.

[B178] Shaban H. Humeau Y. Herry C. Cassasus G. Shigemoto R. Ciocchi S.. (2006). Generalization of amygdala LTP and conditioned fear in the absence of presynaptic inhibition. Nat. Neurosci. 9, 1028–1035. 10.1038/nn1732 16819521

[B179] Shabel S. J. Proulx C. D. Piriz J. Malinow R. (2014). GABA/glutamate co-release controls habenula output and is modified by antidepressant treatment. Science 345, 1494–1498. 10.1126/science.1250469 25237099 PMC4305433

[B180] Shackman A. J. Fox A. S. (2016). Contributions of the central extended amygdala to fear and anxiety. J. Neurosci. 36, 8050–8063. 10.1523/JNEUROSCI.0982-16.2016 27488625 PMC4971357

[B181] Shang C. Liu Z. Chen Z. Shi Y. Wang Q. Liu S.. (2015). A parvalbumin-positive excitatory visual pathway to trigger fear responses in mice. Science 348, 1472–1477. 10.1126/science.aaa8694 26113723

[B182] Shansky R. M. (2018). Sex differences in behavioral strategies: avoiding interpretational pitfalls. Curr. Opin. Neurobiol. 49, 95–98. 10.1016/j.conb.2018.01.007 29414071

[B183] Shields B. C. Kahuno E. Kim C. Apostolides P. F. Brown J. Lindo S.. (2017). Deconstructing behavioral neuropharmacology with cellular specificity. Science 356:eaaj2161. 10.1126/science.aaj2161 28385956

[B184] Soya S. Takahashi T. M. McHugh T. J. Maejima T. Herlitze S. Abe M.. (2017). Orexin modulates behavioral fear expression through the locus coeruleus. Nat. Commun. 8:1606. 10.1038/s41467-017-01782-z 29151577 PMC5694764

[B185] Spence K. W. (1936). The nature of discrimination learning in animals. Psychol. Rev. 43, 427–449. 10.1037/h0056975 14912194

[B186] Stern C. A. J. da Silva T. R. Raymundi A. M. de Souza C. P. Hiroaki-Sato V. A. Kato L.. (2017). Cannabidiol disrupts the consolidation of specific and generalized fear memories via dorsal hippocampus CB1 and CB2 receptors. Neuropharmacology 125, 220–230. 10.1016/j.neuropharm.2017.07.024 28754373

[B187] Sutherland R. J. Rudy J. W. (1989). Configural association theory: the role of the hippocampal formation in learning, memory, and amnesia. Psychobiology 17, 129–144.

[B188] Takehara-Nishiuchi K. Nakao K. Kawahara S. Matsuki N. Kirino Y. (2006). Systems consolidation requires postlearning activation of NMDA receptors in the medial prefrontal cortex in trace eyeblink conditioning. J. Neurosci. 26, 5049–5058. 10.1523/JNEUROSCI.4381-05.2006 16687496 PMC6674241

[B189] Tasan R. O. Verma D. Wood J. Lach G. Hormer B. de Lima T. C.. (2016). The role of Neuropeptide Y in fear conditioning and extinction. Neuropeptides 55, 111–126. 10.1016/j.npep.2015.09.007 26444585

[B190] Temme S. J. Bell R. Z. Pahumi R. Murphy G. G. (2014). Comparison of inbred mouse substrains reveals segregation of maladaptive fear phenotypes. Front. Behav. Neurosci. 8:282. 10.3389/fnbeh.2014.00282 25191238 PMC4139001

[B191] Tolin D. F. Foa E. B. (2006). Sex differences in trauma and posttraumatic stress disorder: a quantitative review of 25 years of research. Psychol. Bull. 132, 959–992. 10.1037/0033-2909.132.6.959 17073529

[B192] Tong M. T. Peace S. T. Cleland T. A. (2014). Properties and mechanisms of olfactory learning and memory. Front. Behav. Neurosci. 8:238. 10.3389/fnbeh.2014.00238 25071492 PMC4083347

[B193] Toufexis D. J. Myers K. M. Bowser M. E. Davis M. (2007). Estrogen disrupts the inhibition of fear in female rats, possibly through the antagonistic effects of estrogen receptor α (ERα) and ERβ. J. Neurosci. 27, 9729–9735. 10.1523/JNEUROSCI.2529-07.2007 17804633 PMC6672956

[B194] Tovote P. Fadok J. P. Lüthi A. (2015). Neuronal circuits for fear and anxiety. Nat. Rev. Neurosci. 16, 317–331. 10.1038/nrn3945 25991441

[B195] Tsien J. Z. Huerta P. T. Tonegawa S. (1996). The essential role of hippocampal CA1 NMDA receptor-dependent synaptic plasticity in spatial memory. Cell 87, 1327–1338. 10.1016/s0092-8674(00)81827-9 8980238

[B196] Vanvossen A. C. Portes M. A. Scoz-Silva R. Reichmann H. B. Stern C. A. Bertoglio L. J.. (2017). Newly acquired and reactivated contextual fear memories are more intense and prone to generalize after activation of prelimbic cortex NMDA receptors. Neurobiol. Learn. Mem. 137, 154–162. 10.1016/j.nlm.2016.12.002 27919830

[B197] Verma D. Tasan R. O. Herzog H. Sperk G. (2012). NPY controls fear conditioning and fear extinction by combined action on Y_1_ and Y_2_ receptors. Br. J. Pharmacol. 166, 1461–1473. 10.1111/j.1476-5381.2012.01872.x 22289084 PMC3401902

[B198] Vetere G. Restivo L. Cole C. J. Ross P. J. Ammassari-Teule M. Josselyn S. A.. (2011). Spine growth in the anterior cingulate cortex is necessary for the consolidation of contextual fear memory. Proc. Natl. Acad. Sci. U S A 108, 8456–8460. 10.1073/pnas.1016275108 21531906 PMC3100962

[B199] Vieira P. A. Corches A. Lovelace J. W. Westbrook K. B. Mendoza M. Korzus E. (2015). Prefrontal NMDA receptors expressed in excitatory neurons control fear discrimination and fear extinction. Neurobiol. Learn. Mem. 119, 52–62. 10.1016/j.nlm.2014.12.012 25615540 PMC7952093

[B200] Vieira P. A. Lovelace J. W. Corches A. Rashid A. J. Josselyn S. A. Korzus E. (2014). Prefrontal consolidation supports the attainment of fear memory accuracy. Learn. Mem. 21, 394–405. 10.1101/lm.036087.114 25031365 PMC4105719

[B201] Walters E. T. Carew T. J. Kandel E. R. (1981). Associative learning in aplysia: evidence for conditioned fear in an invertebrate. Science 211, 504–506. 10.1126/science.7192881 7192881

[B202] Weinberger N. M. (2011). The medial geniculate, not the amygdala, as the root of auditory fear conditioning. Hear. Res. 274, 61–74. 10.1016/j.heares.2010.03.093 20466051 PMC2949681

[B203] Wiltgen B. J. Silva A. J. (2007). Memory for context becomes less specific with time. Learn. Mem. 14, 313–317. 10.1101/lm.430907 17522020

[B204] Wiltgen B. J. Zhou M. Cai Y. Balaji J. Karlsson M. G. Parivash S. N.. (2010). The hippocampus plays a selective role in the retrieval of detailed contextual memories. Curr. Biol. 20, 1336–1344. 10.1016/j.cub.2010.06.068 20637623 PMC2928141

[B205] Winocur G. Moscovitch M. Sekeres M. (2007). Memory consolidation or transformation: context manipulation and hippocampal representations of memory. Nat. Neurosci. 10, 555–557. 10.1038/nn1880 17396121

[B206] Wood S. C. Anagnostaras S. G. (2011). Interdependence of measures in pavlovian conditioned freezing. Neurosci. Lett. 505, 134–139. 10.1016/j.neulet.2011.10.006 22005576 PMC3215911

[B207] Xu W. Südhof T. C. (2013). A neural circuit for memory specificity and generalization. Science 339, 1290–1295. 10.1126/science.1229534 23493706 PMC3651700

[B208] Yokoyama M. Matsuo N. (2016). Loss of ensemble segregation in dentate gyrus, but not in somatosensory cortex, during contextual fear memory generalization. Front. Behav. Neurosci. 10:218. 10.3389/fnbeh.2016.00218 27872586 PMC5097914

[B209] Zhang W. H. Zhou J. Pan H. Q. Wang X. Y. Liu W. Z. Zhang J. Y.. (2017). δ subunit-containing GABA_A_ receptor prevents overgeneralization of fear in adult mice. Learn. Mem. 24, 381–384. 10.1101/lm.045856.117 28716958 PMC5516689

[B210] Zhao M.-G. Toyoda H. Lee Y.-S. Wu L.-J. Ko S. W. Zhang X.-H.. (2005). Roles of NMDA NR2B subtype receptor in prefrontal long-term potentiation and contextual fear memory. Neuron 47, 859–872. 10.1016/j.neuron.2005.08.014 16157280

[B211] Zhu H. Pleil K. E. Urban D. J. Moy S. S. Kash T. L. Roth B. L. (2014). Chemogenetic inactivation of ventral hippocampal glutamatergic neurons disrupts consolidation of contextual fear memory. Neuropsychopharmacology 39, 1880–1892. 10.1038/npp.2014.35 24525710 PMC4059896

